# VDR is a potential prognostic biomarker and positively correlated with immune infiltration: a comprehensive pan-cancer analysis with experimental verification

**DOI:** 10.1042/BSR20231845

**Published:** 2024-04-30

**Authors:** Xuedi Xia, Feng Xu, Dexing Dai, An Xiong, Ruoman Sun, Yali Ling, Lei Qiu, Rui Wang, Ya Ding, Miaoying Lin, Haibo Li, Zhongjian Xie

**Affiliations:** National Clinical Research Center for Metabolic Diseases, Hunan Provincial Key Laboratory of Metabolic Bone Diseases, and Department of Metabolism and Endocrinology, The Second Xiangya Hospital of Central South University, 139 Middle Renmin Road, Changsha 410011, Hunan, China

**Keywords:** Immune infiltration, Pan-cancer, Prognosis, Tumor microenvironment, Vitamin D receptor

## Abstract

The vitamin D receptor (VDR) is a transcription factor that mediates a variety of biological functions of 1,25-dihydroxyvitamin D_3_. Although there is growing evidence of cytological and animal studies supporting the suppressive role of VDR in cancers, the conclusion is still controversial in human cancers and no systematic pan-cancer analysis of VDR is available. We explored the relationships between VDR expression and prognosis, immune infiltration, tumor microenvironment, or gene set enrichment analysis (GSEA) in 33 types of human cancers based on multiple public databases and R software. Meanwhile, the expression and role of VDR were experimentally validated in papillary thyroid cancer (PTC). VDR expression decreased in 8 types and increased in 12 types of cancer compared with normal tissues. Increased expression of VDR was associated with either good or poor prognosis in 13 cancer types. VDR expression was positively correlated with the infiltration of cancer-associated fibroblasts, macrophages, or neutrophils in 20, 12, and 10 cancer types respectively and this correlation was experimentally validated in PTC. Increased VDR expression was associated with increased percentage of stromal or immune components in tumor microenvironment (TME) in 24 cancer types. VDR positively and negatively correlated genes were enriched in immune cell function and energy metabolism pathways, respectively, in the top 9 highly lethal tumors. Additionally, VDR expression was increased in PTC and inhibited cell proliferation and migration. In conclusion, VDR is a potential prognostic biomarker and positively correlated with immune infiltration as well as stromal or immune components in TME in multiple human cancers.

## Introduction

Vitamin D is primarily recognized for its role in regulating calcium homeostasis and bone health. In recent years, increasing number of studies have revealed that vitamin D has a wide range of potential extra-skeletal effects [1–4], such as anti-cancer effects [5]. VDR mediates the biological actions of 1,25-dihydroxyvitamin D_3_. VDR is widely expressed in human tissues such as intestine, kidney, heart, brain, skin, prostate, and ovary [6]. Studies have shown that VDR inhibits tumor progression by suppressing cell stemness in pancreatic cancer and colitis-associated tumorigenesis in colon cancer [7,8]. Knockout of VDR increases carcinogen-induced mammary hyperplasia and tumor progression in the epidermis and lymphoid tissue in mice [9]. These observations suggest that VDR might serve as a tumor suppressor.

Although the results of cytological studies and animal experiments are encouraging [10–12], the results of clinical studies are controversial. A number of clinical studies have demonstrated that higher expression of VDR is positively correlated with better prognosis or low TNM stage in breast cancer, colorectal cancer, papillary thyroid cancer, lung cancer, urothelial bladder cancer, and childhood solid tumors [13–18]. Hypermethylation may lead to underexpression of VDR and is associated with unfavorable outcomes in pediatric adrenocortical tumors [19]. However, other investigators have reported conflicting results. Al-Azhri et al. have reported that VDR expression is not correlated with breast cancer survival outcomes [20]. Choi et al. have shown that higher expression of VDR mRNA is associated with worse clinical outcome in papillary thyroid cancer based on public multi-genomics data [21]. Sahin et al. have found that higher expression of VDR is associated with lower disease-free survival and greater tumor size in superficial transitional cell carcinoma of the bladder [22]. Meanwhile, the relationship between VDR expression and tumor characteristics in some cancers is still unclear. The role of VDR in tumor progression and tumor immunity remains to be elucidated.

Pan-cancer analysis is a new tool for assessing the role of interested genes in cancers [23–25]. To date, no pan-cancer analysis of the relationship between VDR and human cancers has been performed. Previous clinical studies have mostly focused on the association of VDR expression and polymorphism with patients’ survival [26–28]. In the present study, we not only analyzed the relationship between VDR expression and clinical outcomes but also analyzed genetic alterations and the association of VDR expression with immune infiltration, tumor microenvironment and gene set enrichment analysis in 33 types of human cancers based on multiple platforms including the Tumor Immune Estimation Resource, version 2 (TIMER2.0), the Gene Expression Profiling Interactive Analysis, version 2 (GEPIA 2), cBioPortal, Gene Set Cancer Analysis (GSCA), LinkedOmics, the Tumor-immune System Interactions and Drugbank (TISIDB), The University of ALabama at Birmingham CANcer data analysis Portal (UALCAN), and R software. Furthermore, the expression and function of VDR were verified through *in vitro* experiments in PTC. This is the first pan-cancer analysis of VDR and the results provide new insights into the relationship between VDR and various types of cancers, thus may offer comprehensive references for further clinical research.

The 33 types of human cancers included in the present study were listed as follows: adrenocortical carcinoma (ACC), bladder urothelial carcinoma (BLCA), breast invasive carcinoma (BRCA), cervical squamous cell carcinoma and endocervical adenocarcinoma (CESC), cholangiocarcinoma (CHOL), colon adenocarcinoma (COAD), lymphoid neoplasm diffuse large B-cell lymphoma (DLBC), esophageal carcinoma (ESCA), glioblastoma multiforme (GBM), head and neck squamous cell carcinoma (HNSC), kidney chromophobe (KICH), kidney renal clear cell carcinoma (KIRC), kidney renal papillary cell carcinoma (KIRP), acute myeloid leukemia (LAML), lower grade glioma (LGG), liver hepatocellular carcinoma (LIHC), lung adenocarcinoma (LUAD), lung squamous cell carcinoma (LUSC), mesothelioma (MESO), ovarian serous cystadenocarcinoma (OV), pancreatic adeno-carcinoma (PAAD), pheochromocytoma and paraganglioma (PCPG), prostate adenocarcinoma (PRAD), rectum adenocarcinoma (READ), sarcoma (SARC), skin cutaneous melanoma (SKCM), stomach adenocarcinoma (STAD), testicular germ cell tumors (TGCT), thyroid carcinoma (THCA), thymoma (THYM), uterine corpus endometrial carcinoma (UCEC), uterine carcinosarcoma (UCS), and uveal melanoma (UVM).

## Materials and methods

### Differential expression analysis

The TIMER2.0 online tool (http://timer.cistrome.org/) is a comprehensive platform which provides cancer exploration methods [29]. The ‘Gene_DE’ module of ‘Exploration’ was used to analyze the differential expression of VDR between cancers and adjacent normal tissues across all tumors in The Cancer Genome Atlas (TCGA) database. The main algorithm of differential gene analysis was edgeR [30]. The differential expressions between tumors and adjacent tissues were analyzed by the Wilcoxon test. Supplementary data were obtained from the GEPIA 2 platform (http://gepia2.cancer-pku.cn/#index) when normal data were not available for some types of cancers [31]. The ‘Box Plot’ function of ‘Expression DIY’ was used to analyze the differences in VDR expression levels between tumors and adjacent normal tissues of tumors including ACC, DLBC, LAML, LGG, OV, SARC, SKCM, TGCT, THYM, and UCS. The GEPIA 2 platform was used to analyze differential signature score and the signature score was calculated by mean value of log2 (TPM + 1) of gene. The statistical method for differential analysis was one-way ANOVA (|Log_2_FC| Cutoff = 1, *p*-value Cutoff = 0.05).

The cBioPortal for Cancer Genomics (http://www.cbioportal.org/) allows us to present the expression of VDR among different types of human cancers [32]. The ‘Plots’ module was used and the tumor category was sorted according to the median of VDR expression levels. RNA-Seq by Expectation-Maximization (RESM) algorithm was used to analyze mRNA profile. The GSCA database (http://bioinfo.life.hust.edu.cn/GSCA/#/) integrates abundant genomic data across 33 cancer types from TCGA [33]. The ‘Expression’ module was used to investigate the relationship between VDR expression and tumor pathologic stages. The pathologic stage of tumor samples was classified into four stages including stage I, II, III and IV. The differences between the log2 RSEM expression of VDR in 4 stages were analyzed by ANOVA test.

### Survival analysis

Univariate COX regression analysis was used to analyze the relationship between VDR expression and overall survival (OS), progression free survival (PFS), disease specific survival (DSS), or disease free interval (DFI) in various cancers. The results were shown by forest plots based on R packages ‘survival’ and ‘forestplot’ (R software Version 4.1.1). The clinical data were obtained from UCSC Xena database [34] (https://xenabrowser.net/datapages/). The GSCA platform also provides information about clinical outcomes of different VDR expression levels in human cancers [35]. In order to further confirm and more intuitively demonstrate the relationship between VDR expression and survival, the ‘Expression & Survival’ module was used to examine the relationship between VDR expression and OS, PFS, DSS, or DFI in human cancers. Cox Proportional-Hazards model and Logrank tests were used to analyze the specific gene in every cancer.

### Genetic alteration and mutation analysis

The cBioPortal platform was also used to analyze the genetic alterations across all TCGA tumors [32]. The ‘Cancer Types Summary’ module was used to analyze the gene alteration frequency of VDR in multiple cancers. The mutation sites of VDR were visualized in the schematic diagram and 3D structure of protein through ‘Mutation’ function. To further explore the relationship between gene alterations and survival, we searched the name of each cancer with genetic alterations of VDR such as ‘ACC’ under ‘Comparison/Survival’ module. The differences in the survival time between the VDR altered group and the VDR unaltered group were shown.

### Immune infiltration analysis

The correlations between VDR expression and abundance of multiple immune infiltrates including natural killer (NK) cells, CD4+ T cells, CD8+ T cells, T regulatory cells (Tregs), B cells, cancer as-sociated fibroblasts, macrophages, neutrophils, myeloid dendritic cells, and monocytes in various tumors were analyzed by the ‘Gene’ function under ‘Immune’ module in the TIMER2.0 platform [29]. The heatmaps based on purity-adjusted Spearman’s rho were generated to show the association between VDR expression and immune infiltration. Multiple algorithms including EPIC, MCPCOUNTER, QUANTISEQ, XCELL, CIBERSORT, CIBERSORT-ABS, TIMER and TIDE were used to estimate immune infiltration levels.

### Immune-related gene analysis

The TISIDB platform (http://cis.hku.hk/TISIDB/) offers a plentiful resource for tumor-immune system interaction [36]. Spearman correlations between VDR expression and immune-related genes including immunoinhibitor, immunostimulator, MHCs, chemokine, or chemokine receptor were analyzed. A linear plot was generated after a single cancer and an immune modulator were chosen.

### Tumor microenvironment analysis

The association between VDR expression and TME related pathway scores in various tumors was analyzed by Pearson’s correlation and the result was shown by heatmap based on R software (Version 4.1.1). The TME related pathway score was established by the principal component analysis algorithms [37]. StromalScore (the components of stromal cells), ImmuneScore (the components of immune cells), ESTIMATEScore (the sum of StromalScore and ImmuneScore), and TumorPurity were calculated by R package ‘ESTIMATE’ algorithm [38]. The correlation between VDR expression and ESTIMATE analysis was computed via Pearson’s correlation and visualized by heatmap and lollipop-diagrams. The data used for analysis were obtained from UCSC Xena database [34].

### Gene set enrichment analysis

The LinkedOmics platform (http://www.linkedomics.org/admin.php) provides available multi-omics data from TCGA database and 10 Clinical Proteomics Tumor Analysis Consortium (CPTAC) cancer cohorts [39]. In order to probe into biological insights from the correlation, the ‘LinkInterpreter’ module of the LinkedOmics database was used to perform enrichment analysis for the top 9 tumors in terms of estimated deaths based on cancer statistics published in 2022 [40]. Gene set enrichment analysis was performed for Kyoto Encyclopedia of Genes and Gnomes (KEGG) pathway enrichment analysis. The enrichment analysis was based on Pearson correlation test and the results were displayed in bar graphs.

### Cell culture

The PTC cell lines K1 and BCPAP were respectively purchased from American Type Culture Collection (ATCC) and China Center for Type Culture Collection (CCTCC). The human normal thyroid cell line Nthy-ori-3-1 was obtained from Cell Bio, Shanghai, China. All three cell lines were identified by STR profiling. K1 cells were cultured in high-glucose (4.5 g/L) Dulbecco's Modified Eagle Medium (DMEM), BCPAP cells were cultured in RPMI-1640 medium and Nthy-ori-3-1 cells were cultured in DMEM, nutrient mixture F-12 supplemented with 10% fetal bovine serum (FBS, Biological Industries, Israel), 100 units/ml penicillin and 100 μg/ml streptomycin (Biological Industries, Israel). All cells were cultured in an incubator maintained in 37°C and 5% CO_2_.

### Lentivirus infection

A total number of 1 × 10^5^ K1 and BCPAP cells were plated in 6-well plates. K1 and BCPAP cells were transduced the lentiviral vector containing the puromycin resistance gene and VDR-shRNA or Control-shRNA (Genechem, Shanghai, China) at the multiplicity of infection (MOI) of 50 or 10 respectively for 16 h. After the cells were cultured in the complete medium for 72 h, the green fluorescence was observed to evaluate transfection efficiency under a luminescence microscope (Carl Zeiss Microscopy GmbH, Germany). Stably infected cells were selected by puromycin (Genechem, Shanghai, China) for 2–3 days.

### Adenovirus transfection

K1 and BCPAP cells were seeded in 6-well plates at 3–5 × 10^5^/well. Cells were infected with adenovirus vector containing human VDR cDNA (VDR-adv) (Genechem, Shanghai, China) or negative control adenovirus (Control-adv) at MOI of 100 in the presence of enhanced infection solution (Genechem, Shanghai, China) for 12 h. Green fluorescence was observed after virus-infected cells were cultured in the complete medium for 72 h.

### Patients and tissue samples

PTC tissues and matched adjacent normal tissues were obtained from patients who underwent thyroidectomy at the Second Xiangya Hospital of Central South University. Specimens from 10 patients were embedded in paraffin and used for immunohistochemistry. Specimens from other 27 patients were snap-frozen in liquid nitrogen and used for the extraction of tissue RNA and protein. The study was approved by the ethics committee in the Second Xiangya Hospital of Central South University. All patients have signed a written informed consent form.

### Western blot analysis

The UALCAN database (http://ualcan.path.uab.edu/) was used to examine the expression levels of VDR according to different histology in thyroid carcinoma. The expression of VDR in PTC cell lines and tissues was further verified by Western blot analysis. Detailed procedures of Western blot analysis were performed as described previously [41]. The antibodies used were shown as follows: antibodies against VDR (D-6, sc-13133, Santa Cruz Biotechnology, 1:500), GAPDH (10494-1-AP, Proteintech, 1:3000), HRP-conjugated affinipure goat anti-mouse IgG(H+L) (SA00001-1, Proteintech, 1:5000), HRP-conjugated affinipure goat anti-rabbit IgG(H+L) (SA00001-2, Proteintech, 1:5000).

### Quantitative real-time polymerase chain reaction (qRT-PCR)

Total RNA was isolated from virus-infected K1 and BCPAP cells as well as PTC tissues and matched adjacent normal tissues using Trizol reagent (Invitrogen, U.S.A.) and reverse transcribed into cDNA by PrimeScript™ RT reagent Kit with gDNA Eraser (RR047A, Takara, Japan). qRT-PCR was subsequently performed using TB Green® Premix Ex Taq™ II (RR820A, Takara, Japan) as described previously [42]. The mRNA expression levels of VDR were quantified by the 2^−ΔΔCq^ method. The average *C*q values of VDR and fibroblast activation protein (FAP), CD68 or CD15 of the PTC tissues were used for correlation analysis. The primers used for qRT-PCR were listed below: VDR: forward primer 5′-TCTCCAATCTGGATCTGAGTGAA-3′ and reverse primer 5′-GGATGCTGTAACTGACCAGGT-3′, GAPDH: forward primer 5′- TGATGACATCAAGGTGGTGAAG-3′ and reverse primer 5′- TCCTTGGAGGCCATGTGGGCCAT-3′, FAP: forward primer 5′-ATGAGCTTCCTCGTCCAATTCA-3′ and reverse primer 5′-AGACCACCAGAGAGCATATTTTG-3′, CD68: forward primer 5′- GGAAATGCCACGGTTCATCCA-3′ and reverse primer 5′-TGGGGTTCAGTACAGAGATGC-3′, CD15: forward primer 5′- GATCTGCGCGTGTTGGACTA-3′ and reverse primer 5′-GAGGGCGACTCGAAGTTCAT-3′.

### Immunohistochemistry (IHC)

The human thyroid cancer specimens were obtained from 10 patients with PTC at the Second Xiangya Hospital of Central South University in China. The paraffin-embedded cancer tissues were cut into 4-μm thick sections. Tissue sections were dewaxed in xylene and rehydrated in ethanol with a gradually decreased concentration. Antigen retrieval was conducted by 0.25% trypsin with EDTA for 30 min at 37°C and then endogenous peroxidase activity was blocked by 0.3% hydrogen peroxide for 20 min at room temperature. Sections were blocked with 5% bovine serum albumin for 1 h and incubated with the primary antibody for VDR (1:50 dilution, sc-13133, Santa Cruz Biotechnology) overnight at 4°C. After washing three times with phosphate-buffered saline the next day, the sections were incubated the secondary antibody for 30 min at 37°C (PV-9000, ZSGB-BIO, China). Subsequently, the slides were stained separately by 3,3'-diaminobenzidine (DAB, ZLI-9018, ZSGB-BIO, China) and hematoxylin (G1004, Servicebio, China). Images were taken by a microscope (Olympus CX31, Tokyo, Japan) magnified ×400. ImageJ software (National Institutes of Health, the United States) was used to calculate average optical density (AOD, integrated option density/ area). The average AOD of the five visual fields was used for analysis.

### Cell counting kit-8 (CCK-8) assay

K1 and BCPAP cells transfected by lentivirus and adenovirus were seeded into 96-well plates at a density of 2 × 10^3^ cells per well in 100 µl complete medium. Each group had four replicates. After cells were cultured for 0, 24, 48, 72 and 96 h, 10 µl CCK-8 reagent (BS350B, Biosharp, China) and 90 µl basic medium were added into each well and incubated for 2 h at 37 °C. The optical density value (OD value) was measured at 450 nm by a microplate reader (Bio-Tek Instruments, Inc., U.S.A.).

### Colony formation assay

After transfection with the lentivirus and adenovirus, K1 and BCPAP cells were seeded into 6-well plates (1000 cells/ well) and cultured for 14 days. The culture medium was changed every 2–3 days. The colony numbers were analyzed by ImageJ software (National Institutes of Health, the United States) after the cells were fixed with 4% paraformaldehyde and stained with 0.1% crystal violet solution for 10 min.

### Transwell migration assay

Transfected K1 and BCPAP cells were seeded into the upper chamber at 5 × 10^4^ in 200 μl serum-free medium and 600 μl complete medium (10% FBS) were added into the lower chamber of Transwell plates (3422, Corning, pore size: 8.0 μm). After the cells were subsequently incubated at 37°C for 48 h, the membranes were fixed with 4% paraformaldehyde and stained with 0.1% Crystal Violet for 10 min. The cells remaining on the upper membrane were removed gently with cotton swabs and migration cells were observed and photographed using a light microscope (Olympus CX31, Tokyo, Japan). Migration cells in three fields of each chamber were counted by ImageJ software (National Institutes of Health, U.S.A.).

### Statistical methods

GraphPad Prism version 7.0 (GraphPad Software, U.S.A.) was used for data analysis. Experimental data were presented as mean ± standard deviation (SD) and compared by the Student’s *t*-test or analysis of variance (ANOVA) and simple linear regression was performed to calculate the correlation. *P*<0.05 was considered statistically significant.

## Results

### VDR expression was decreased in 8 types and increased in 12 types of cancer when compared with adjacent normal tissues

**Figure 1:**
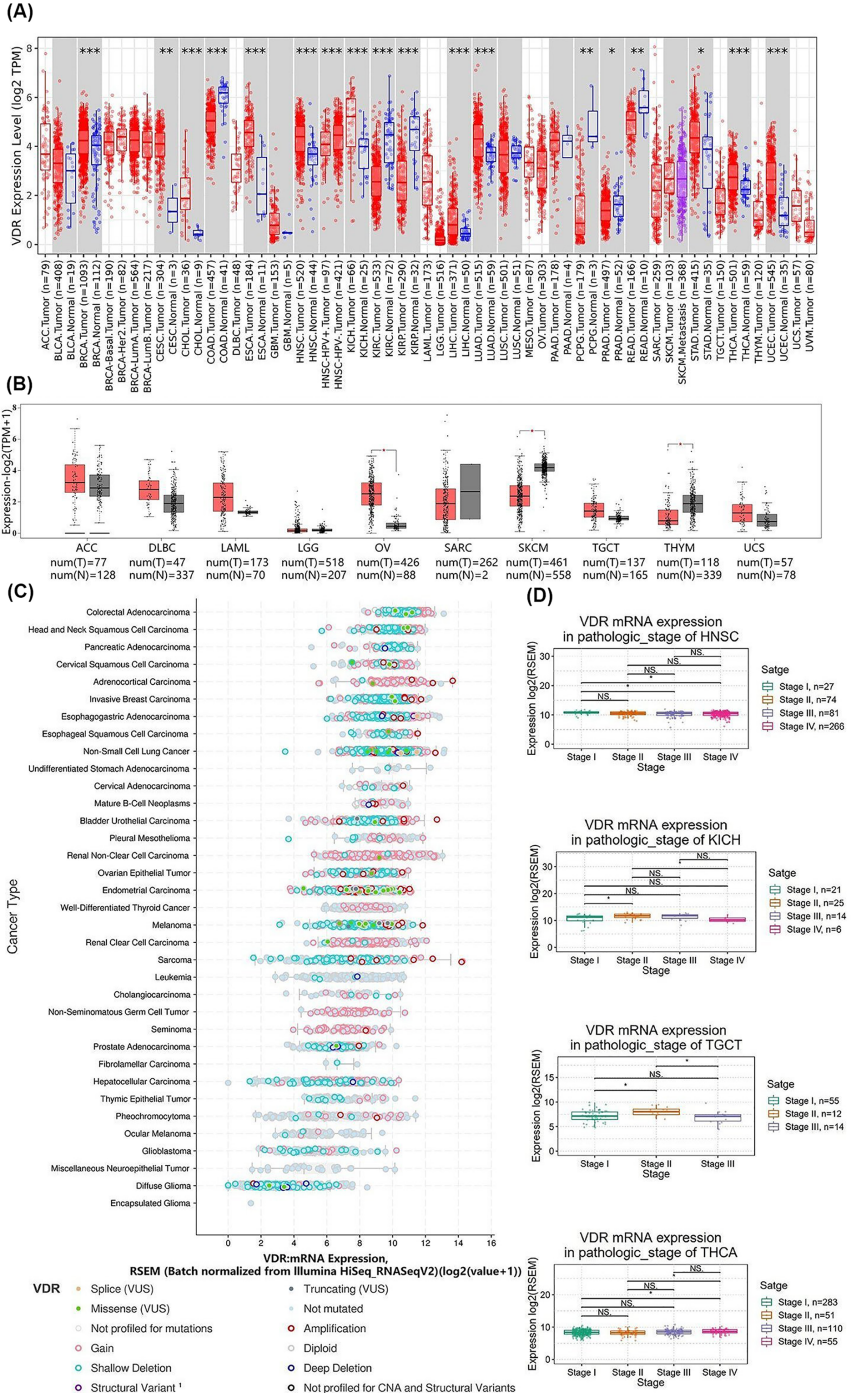
VDR expression was decreased in 8 types and increased in 12 types of cancer when compared with adjacent normal tissues (**A**) The differential expression of VDR between tumors and adjacent normal tissues was provided through the TIMER2.0 platform; *P<0.05, **P<0.01, ***P<0.001. (**B**) For some types of cancers, normal data were matched via TCGA and GTEx databases by the GEPIA 2 platform; *P<0.05. (**C**) The expression levels of VDR in human cancers were showed by the cBioPortal tool. The tumor category was sorted according to the median of VDR expression levels. (**D**) The relationships between VDR expression and pathologic stages of HNSC, KICH, TGCT, or THCA were analyzed based on the GSCA platform; *P<0.05.

We first analyzed differential expression of VDR between tumors and adjacent normal tissues across all tumors in TCGA database. The abbreviations and full names of the 33 types of human cancers included in the present study and changes of expression in these cancers were provided in Table 1. When compared with matched normal tissues, the expression levels of VDR were lower in COAD, KIRC, KIRP, PCPG, PRAD, and READ and higher in tumor tissues of BRCA, CESC, CHOL, ESCA, HNSC, KICH, LIHC, LUAD, STAD, THCA, and UCEC (Figure 1A). For some types of tumors including ACC, DLBC, LAML, LGG, OV, SARC, SKCM, TGCT, THYM, and UCS, normal tissue data were matched from TCGA and genotype-tissue expression (GTEx) databases. As shown in Figure 1B, VDR was lower expression in SKCM, THYM and higher expression in OV than adjacent normal tissues. However, the data of VDR expression in MESO and UVM were not available and not shown.

**Table 1:** The abbreviations and full names of 33 types of human cancers and changes of VDR expression in these cancers

Abbreviations	Full names	Changes of expression
ACC	Adrenocortical carcinoma	→
BLCA	Bladder urothelial carcinoma	→
BRCA	Breast invasive carcinoma	↑
CESC	Cervical squamous cell carcinoma and endocervical adenocarcinoma	↑
CHOL	Cholangiocarcinoma	↑
COAD	Colon adenocarcinoma	↓
DLBC	Lymphoid neoplasm diffuse large B-cell lymphoma	→
ESCA	Esophageal carcinoma	↑
GBM	Glioblastoma multiforme	→
HNSC	Head and neck squamous cell carcinoma	↑
KICH	Kidney chromophobe	↑
KIRC	Kidney renal clear cell carcinoma	↓
KIRP	Kidney renal papillary cell carcinoma	↓
LAML	Acute myeloid leukemia	→
LGG	Brain lower grade glioma	→
LIHC	Liver hepatocellular carcinoma	↑
LUAD	Lung adenocarcinoma	↑
LUSC	Lung squamous cell carcinoma	→
MESO	Mesothelioma	
OV	Ovarian serous cystadenocarcinoma	↑
PAAD	Pancreatic adenocarcinoma	→
PCPG	Pheochromocytoma and paraganglioma	↓
PRAD	Prostate adenocarcinoma	↓
READ	Rectum adenocarcinoma	↓
SARC	Sarcoma	→
SKCM	Skin cutaneous melanoma	↓
STAD	Stomach adenocarcinoma	↑
TGCT	Testicular germ cell tumors	→
THCA	Thyroid carcinoma	↑
THYM	Thymoma	↓
UCEC	Uterine corpus endometrial carcinoma	↑
UCS	Uterine carcinosarcoma	→
UVM	Uveal melanoma	

The up, down, and horizontal arrows indicate increased, decreased, and comparable expression levels when compared with matched normal tissues.

Since VDR is widely expressed in human tissues, we next examined VDR expression in various types of cancers. As shown in Figure 1C, VDR was also widely expressed in human cancers especially in tumors of digestive system and endocrine system. The relationship between VDR expression and pathologic stages of various tumors was analyzed by ANOVA test. As shown in Figure 1D, the expression of VDR varied between different pathological stages of the following tumors: HNSC (*P*<0.01; FDR = 0.09), KICH (*P*<0.05; FDR = 0.35), TGCT (*P*<0.05; FDR = 0.10) and THCA (*P* 0.05; FDR = 0.13). Interestingly, the expression of VDR was increased in early stage (from stage I to stage II) but decreased in late stage (from stage II to stage III or IV) in KICH and TGCT. No association was observed between VDR expression and pathologic stages in other tumors such as CHOL and ACC (Supplementary Figure S1).

### Increased expression of VDR was associated with either good prognosis in CHOL, SKCM, KIRC, ESCA, and OV or poor prognosis in LGG, GBM, LAML, KIRP, UVM, PAAD, ACC, and THCA

**Figure 2:**
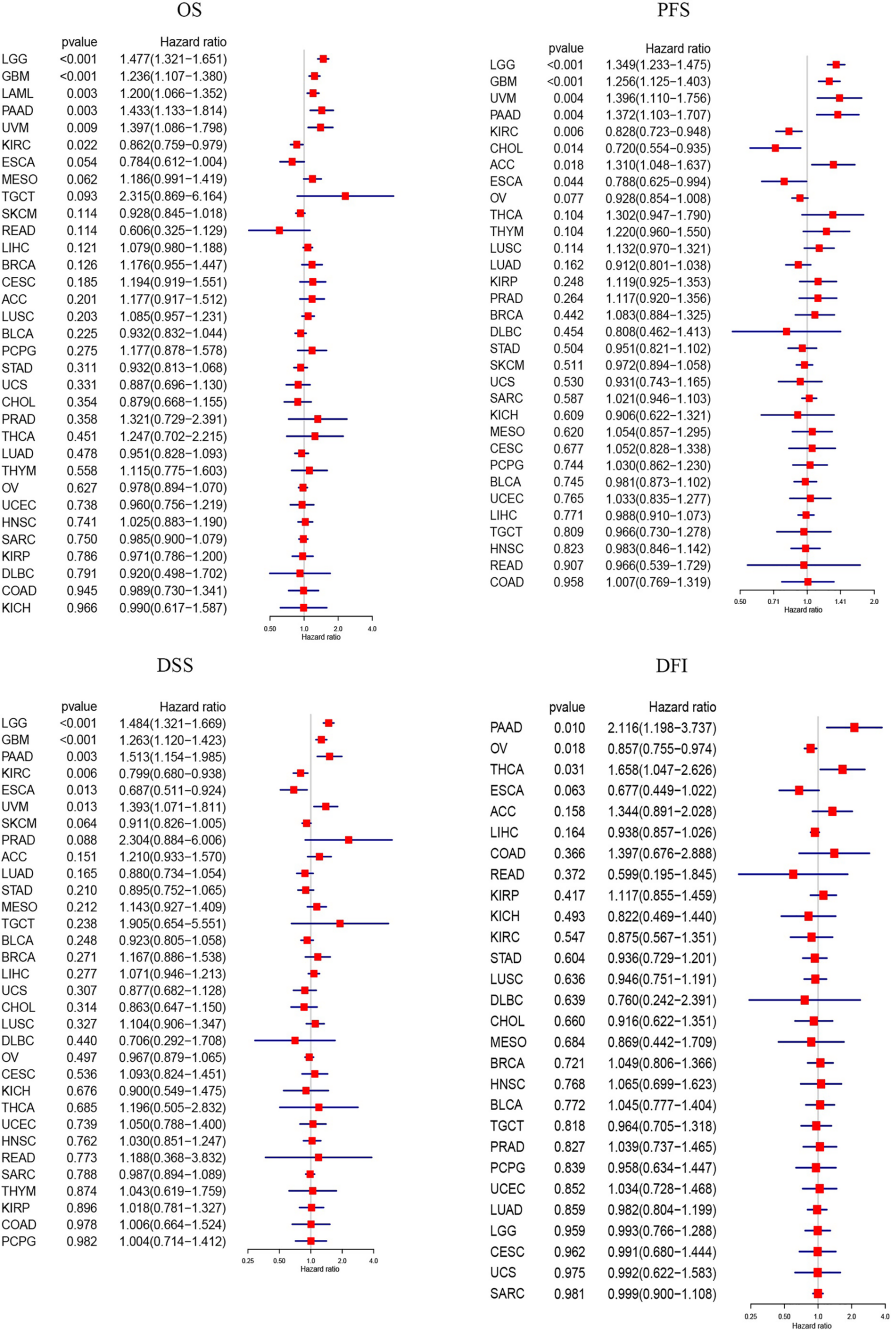
Increased expression of VDR was associated with either good prognosis in CHOL, KIRC, ESCA, and OV or poor prognosis in LGG, GBM, LAML, UVM, PAAD, ACC, and THCA The forest plots were used to show the results of univariate COX regression analysis of OS, PFS, DSS, and DFI via R software. Hazard ratio <1 indicated the protective role of VDR expression for death. *P* value <0.05 was considered statistically significant.

In order to determine the association between VDR expression levels and clinical outcomes, we analyzed the relationship between VDR expression and OS, PFS, DSS, or DFI by R software. As shown in Figure 2, higher expression of VDR was associated with better OS in KIRC, better PFS in KIRC, CHOL and ESCA, better DSS in KIRC and ESCA, or better DFI in OV. However, lower expression of VDR was associated with better OS in LGG, GBM, LAML, PAAD, and VUM. The results of PFS analysis showed that higher expression of VDR was associated with worse prognosis in patients with LGG, GBM, UVM, PAAD, and ACC. Furthermore, VDR expression was negatively correlated with DSS in LGG, GBM, PAAD, UVM and DFI in PAAD or THCA. In addition, we conducted survival analysis of OS, PFS, DSS, and DFI across 33 cancer types from TCGA database based on the GSCA platform. As demonstrated in Supplementary Figure S2, the results were consistent with the data of OS in LGG, GBM, LAML, PAAD, and UVM and the data of PFS in LGG, GBM, and UVM which were analyzed by R software. Besides, higher expression of VDR was associated with better OS, PFS, DSS, and DFI in CHOL and better DSS in SKCM but poor PFS in KIRP. To sum up, increased expression of VDR was associated with good prognosis in CHOL, SKCM, KIRC, ESCA, and OV but poor prognosis in LGG, GBM, LAML, KIRP, UVM, PAAD, ACC, and THCA.

### VDR genetic alterations and negative association with survival in ACC, LUAD, and OV

**Figure 3:**
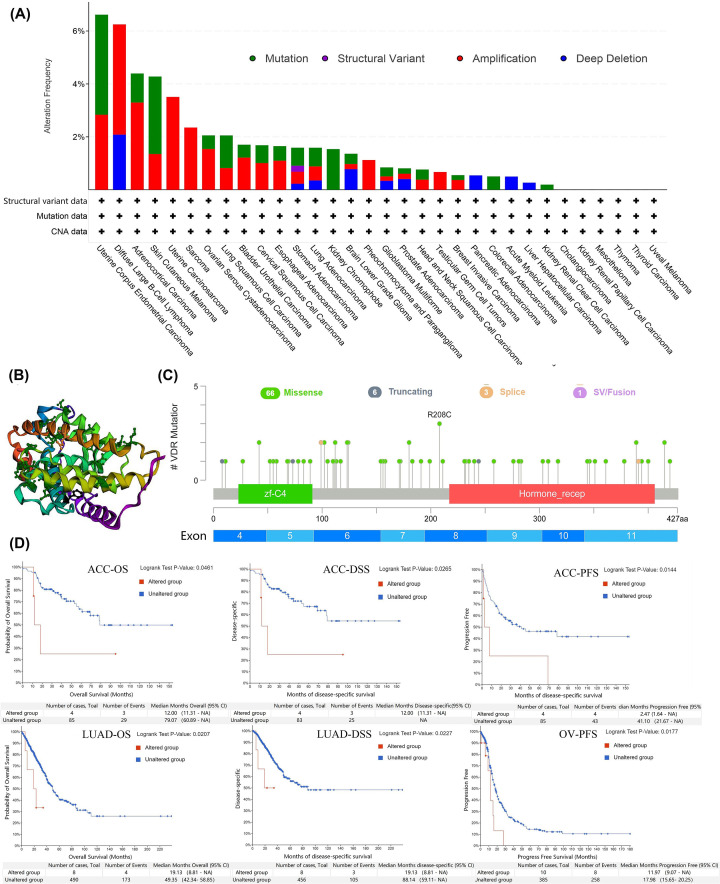
VDR genetic alterations and negative association with survival in ACC, LUAD and OV Alteration related data were obtained through the cBioPortal platform based on TCGA database. (**A**) The frequency of alterations involving missense, truncating, spice and SV/fusion of VDR in various human tumors, (**B**) the mutation site in the 3D structure and (**C**) schematic diagram of protein were displayed. (**D**) The negative relationships between the genetic alterations of VDR and OS, DSS, or PFS in ACC, LUAD, and OV were showed. *P* value <0.05 was considered statistically significant.

Genetic alterations which are frequently occurred in various tumors are thought to participate in cancer initiation and progression [43]. Therefore, we explored genetic alterations of VDR in TCGA pan-cancer atlas. The alteration types included mutation, structural variant, amplification, and deep deletion in various cancers, among which amplification was the most common type (Figure 3A). The highest alteration frequency (>6%), including VDR mutation and amplification, occurred in uterine corpus endometrial carcinoma. All alteration types of VDR were found in stomach adenocarcinoma and amplification of VDR was observed in all uterine carcinosarcoma and sarcoma. No genetic alteration was found in some types of tumors including cholangiocarcinoma, kidney renal papillary cell carcinoma, mesothelioma, thymoma, thyroid carcinoma, and uveal melanoma. The mutation sites of VDR protein were shown in 3D structure (Figure 3B) and schematic diagram (Figure 3C). As shown in the figure, mutations of VDR were composed of missense, truncating, splice and SV/Fusion, among which the missense mutation was the major type. Thereinto, the mutation site R208C with the highest alteration frequency was found in astrocytoma and uterine endometrioid carcinoma. Furthermore, we analyzed the correlations between VDR genetic alterations and the clinical outcomes across multiple tumors. The results with statistical differences between altered group and unaltered group of VDR were shown in Figure 3D. The altered group of VDR showed poor OS in ACC (*P*=0.0461) and LUAD (*P*=0.0207), poor DSS in ACC (*P*=0.0265) and LUAD (*P*=0.0227), or poor PFS in ACC (*P*=0.0144) and OV (*P*=0.0177).

### Positive relationships between VDR expression and immune infiltration of cancer-associated fibroblasts, macrophages or neutrophils in 20, 12, and 10 types of human tumors, respectively

**Figure 4:**
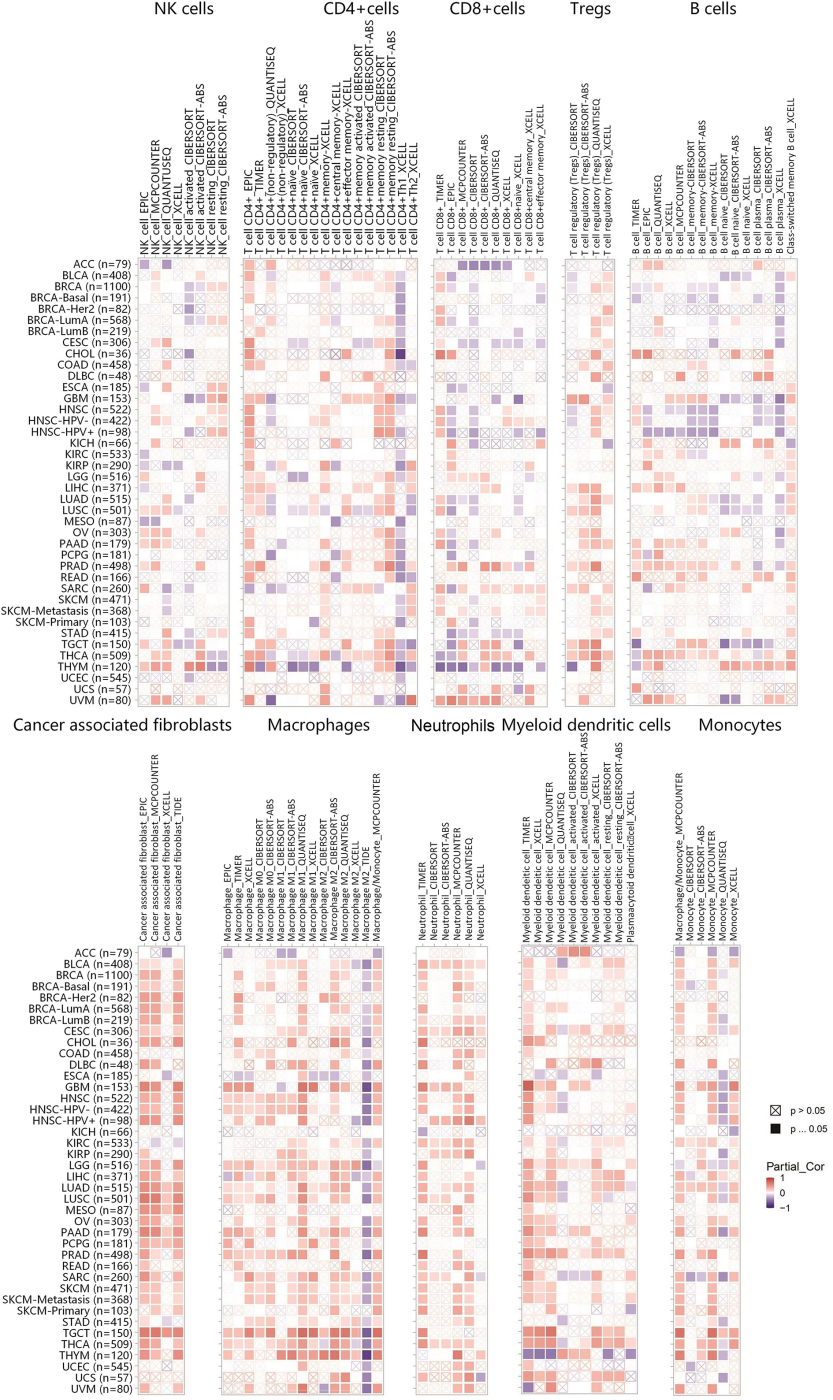
Positive correlations between VDR expression and immune infiltration of cancer-associated fibroblasts, macrophages or neutrophils in 20, 12, and 10 types of human tumors respectively The correlations between VDR expression and immune infiltration levels covering NK cells, CD4+ T cells, CD8+ T cells, Tregs, B cells, cancer-associated fibroblasts, macrophages, neutrophils, myeloid cells, or monocytes were showed by heatmaps based on the TIMER2.0 platform. The red color indicates positive correlations while the blue color indicates negative correlations. *P* value <0.05 was considered statistically significant.

We analyzed the relationships between VDR expression and immune infiltration levels of NK cells, CD4+ T cells, CD8+ T cells, Tregs, B cells, cancer-related fibroblasts, macrophages, neutrophils, myeloid dendritic cells, and monocytes in various cancer types in TCGA database. The correlation between immune infiltration and VDR expression was analyzed by diverse algorithms like TIMER, EPIC, and XCELL. As shown in Figure 4, VDR expression was positive correlated with infiltration of cancer associated fibroblasts in BLCA, BRCA, CESC, CHOL, GBM, HNSC, LGG, LIHC, LUAD, LUSC, MESO, OV, PAAD, PCPG, PRAD, SARC, SKCM, TGCT, THCA, and THYM and infiltration of macrophages in GBM, HNSC, LGG, LUAD, LUSC, PAAD, PRAD, SARC, SKCM, TGCT, THCA, and THYM and infiltration of neutrophils in BLCA, CESC, COAD, GBM, KIRC, KIRP, LUAD, LUSC, SARC, and THCA based on all or more than half of the algorithms. Furthermore, a positive correlation between VDR expression and infiltration of CD8+ T cells in UVM and infiltration of Tregs in THCA was also shown in Figure 4.

### Positive associations between VDR expression and various immune-related genes especially in TGCT and GBM

**Figure 5:**
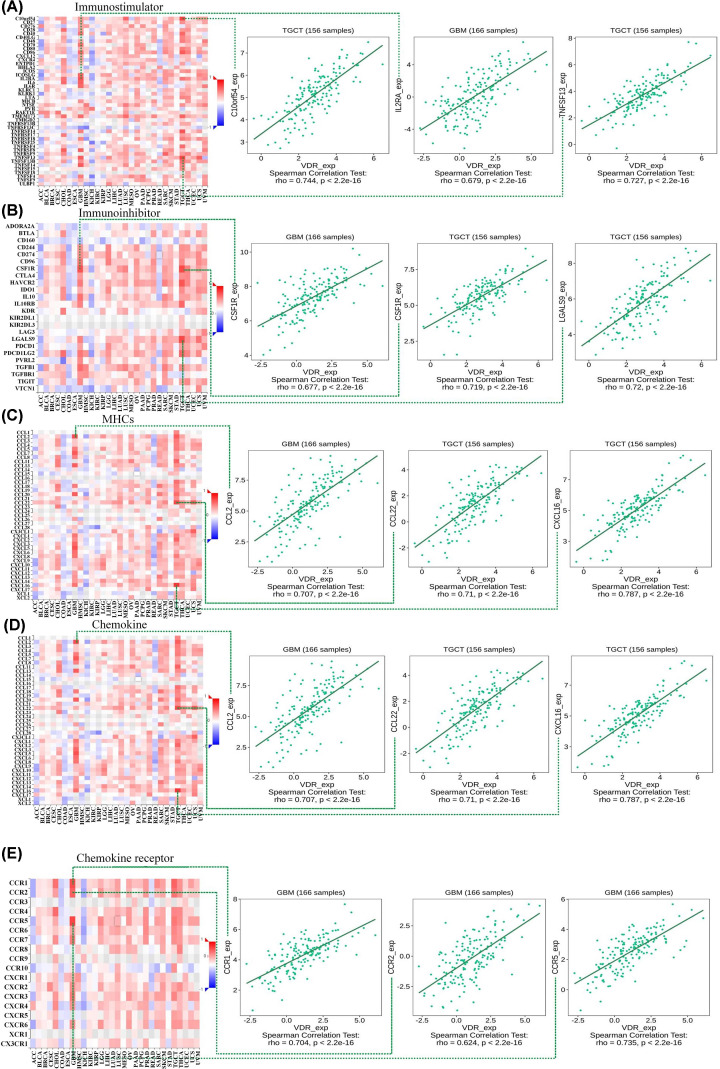
Positive associations between VDR expression and various immune-related genes in human tumors especially in TGCT and GBM We used the TISIDB online tool to explore the correlations between VDR expression and (**A**) immunostimulator, (**B**) immunoinhibitor, (**C**) MHCs, (**D**) chemokine, or (**E**) chemokine receptor. The red color indicates positive correlations while the blue color indicates negative correlations. The top 3 highest values of Spearman’s correlation were shown in scatter plots.

We analyzed the interaction between VDR expression and immune-related genes in TCGA database. Correlations between VDR expression and numerous immunomodulators were shown in Figure 5. The rho values of Spearman’s correlation were provided in Supplementary Table S1. The value greater than 0 indicated a positive correlation while <0 indicated a negative correlation. The top 3 strongest correlations were presented by scatter diagrams. The expression of VDR was positively correlated with C10orf54, tumor necrosis factor TNF receptor superfamily member 13 (TNFRSF13) in TGCT or interleukin-2 α-chain receptor (IL2RA) in GBM for immunostimulators (Figure 5A). Positive correlations between VDR expression and immunoinhibitors of colony stimulating factor 1 receptor (CSF1R), galectin-9 (LGALS9) in TGCT, or GSF1R in GBM were shown in Figure 5B. Besides, VDR expression was positively associated with major histocompatibility complexes (MHCs) of β2-microglobulin (B2M), human leukocyte antigen-A (HLA-A) and tapasin-binding protein (TAPBP) in TGCT (Figure 5C). As for chemokine and chemokine receptor, VDR expression was positively correlated with C-C motif chemokine ligand 2 (CCL2), C-C motif chemokine receptor 1 (CCR1), C-C motif chemokine receptor 2 (CCR2), and C-C motif chemokine receptor 5 (CCR5) in GBM or C-C chemokine ligand 22 (CCL22), C-X-C motif chemokine ligand 16 (CXCL16) in TGCT (Figure 5D,E). Positive correlations between VDR expression and a great number of immunomodulators were also shown in Figure 5. The results suggest that increased expression of VDR is associated with increased expressions of various immune-related genes in multiple tumors particularly in GBM and TGCT.

### Positive correlations between VDR expression and proportion of stromal or immune components in tumor microenvironment in 24 types of human cancers

**Figure 6:**
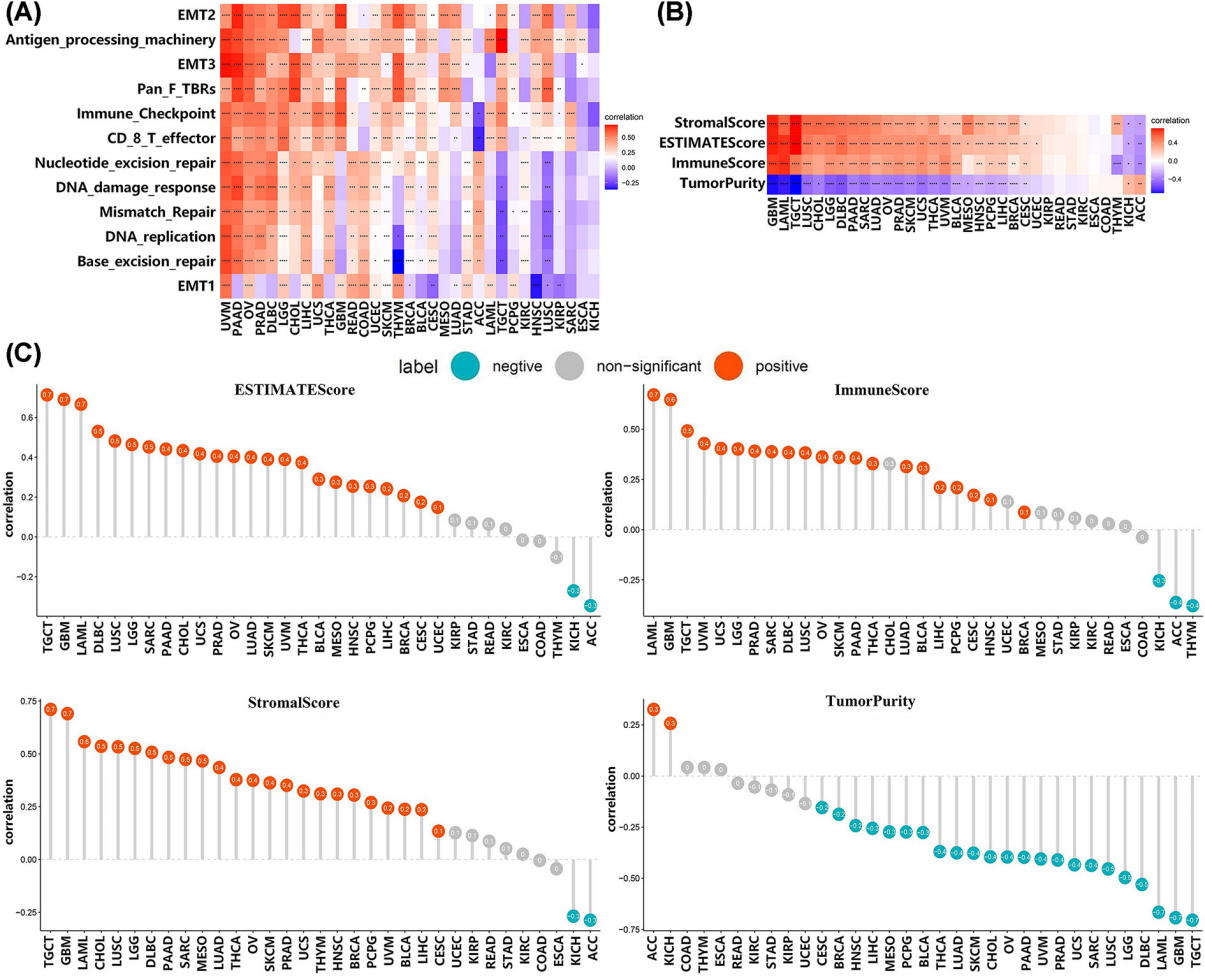
VDR expression was positively correlated with increased proportion of stromal or immune components in tumor microenvironment in 24 types of human cancers (**A**) The correlation between VDR expression and TME related pathway scores was shown; *P<0.05, **P<0.01, ***P<0.001, ****P<0.0001. The correlations between VDR expression and StromalScore, ImmuneScore, ESTIMATEScore, or TumorPurity of tumor tissues were visualized by (**B**) heatmap and (**C**) lollipop-diagrams.

Tumor microenvironment is thought to be potential therapeutic target in many types of cancers in recent years, thus we analyzed the correlation between VDR expression and tumor microenvironment. As shown in Figure 6A, VDR expression was positively associated with TME-related pathways including antigen processing machinery and CD8+ T effector as well as nucleotide excision repair, DNA damage response and base excision repair in various tumors such as UVM, PAAD, OV, PRAD, and DLBC. A strong positive correlation between VDR expression and antigen processing machinery in TGCT was observed. In addition, VDR expression had a positive relationship with StromalScore, ESTIMATEScore or ImmuneScore while was negatively correlated with TumorPurity in TGCT, GBM, LAML, DLBC, LUSC, LGG, SARC, PAAD, CHOL, UCS, PRAD, OV, LUAD, SKCM, UVM, THCA, BLCA, MESO, HNSC, PCPG, LIHC, BRCA, CESC, and UCEC (Figure 6B,C). The results indicated that high VDR expression was related to high stromal components or immune components in the aforementioned tumors. These data reveal that VDR may play a significant role in regulating tumor microenvironment.

### VDR positively and negatively correlated genes were enriched in immune cell function and energy metabolism pathways respectively in the top 9 highly lethal tumors

**Figure 7:**
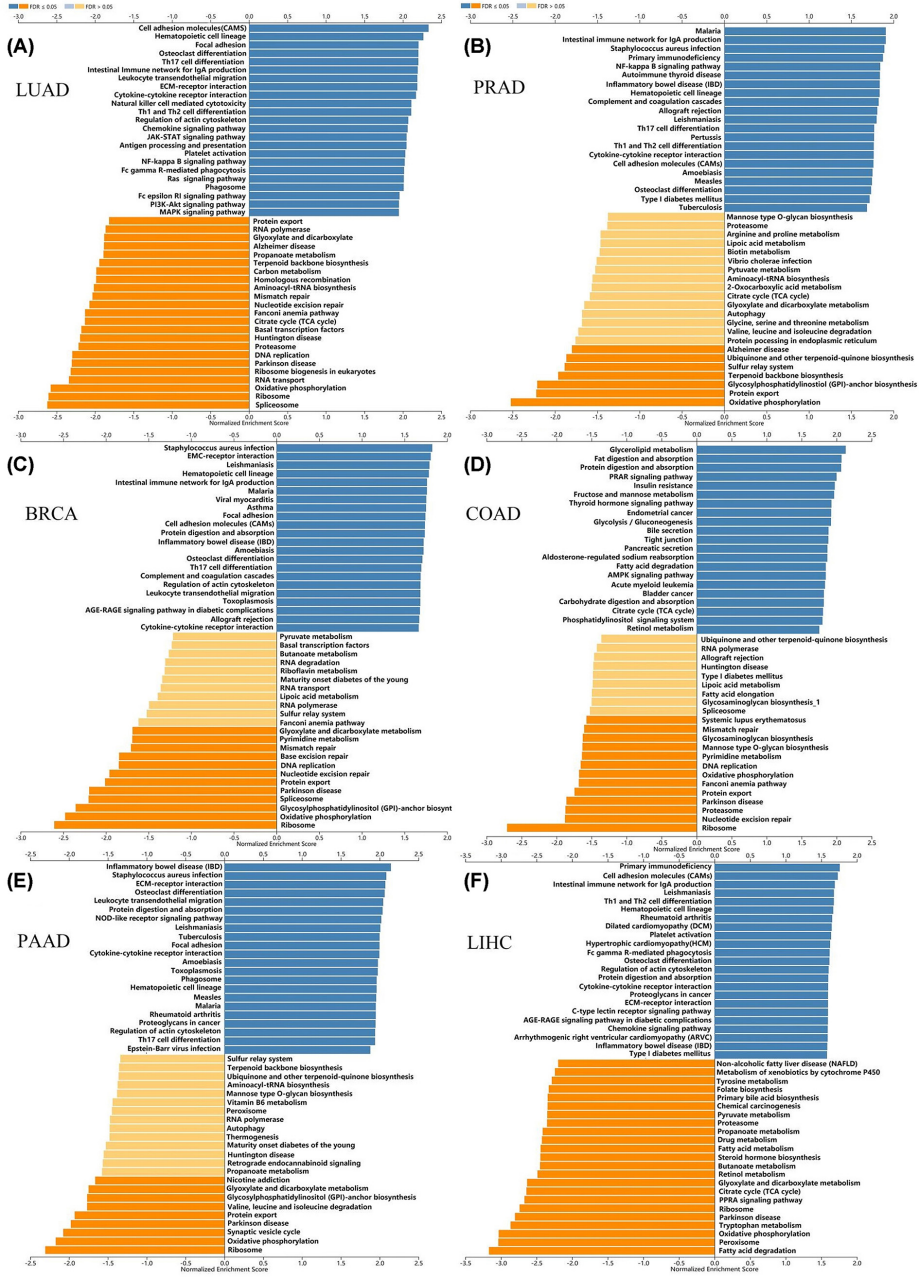
VDR positively and negatively correlated genes were enriched in immune cell function and energy metabolism pathways respectively in 6 types of highly lethal tumors VDR related KEGG pathway for 6 types of highly lethal tumors ranked by estimated deaths were analyzed by the LinkedOmics database and were shown as follows: (**A**) LUAD, (**B**) PRAD, (**C**) BRCA, (**D**) COAD, (**E**) PAAD, and (**F**) LIHC. The blue color indicates positive related categories (FDR ≤ 0.05), the orange color indicates negative related categories (FDR ≤ 0.05) while the yellow color represents no related categories. Horizontal axis indicates normalized enrichment score.

We also conducted GSEA analysis to further explore VDR related KEGG pathways in the top 9 tumors with high estimated mortality rates. The correlations between VDR expression and co-expressed gene related KEGG pathways in LUAD, PRAD, BRCA, COAD, PAAD, LIHC, OV, LAML, and UCEC were analyzed. As shown in Figure 7A–F and Supplementary Figure S3, VDR positively associated genes were enriched in immune related pathways such as Th1, Th2 differentiation in LUAD, PRAD, LIHC, OV, UCEC, and cytokine–cytokine receptor interaction in PRAD, PAAD, OV, and UCEC. On the contrary, VDR negatively associated genes were involved in energy metabolism related pathways such as glyoxylate and dicarboxylate metabolism in BRCA and PAAD as well as oxidative phosphorylation in PRAD, COAD, PAAD, LIHC, and UCEC.

### VDR expression was up-regulated in PTC and the expression of VDR was positively correlated with the expression of cancer-associated fibroblast, macrophage and neutrophil markers in PTC

**Figure 8:**
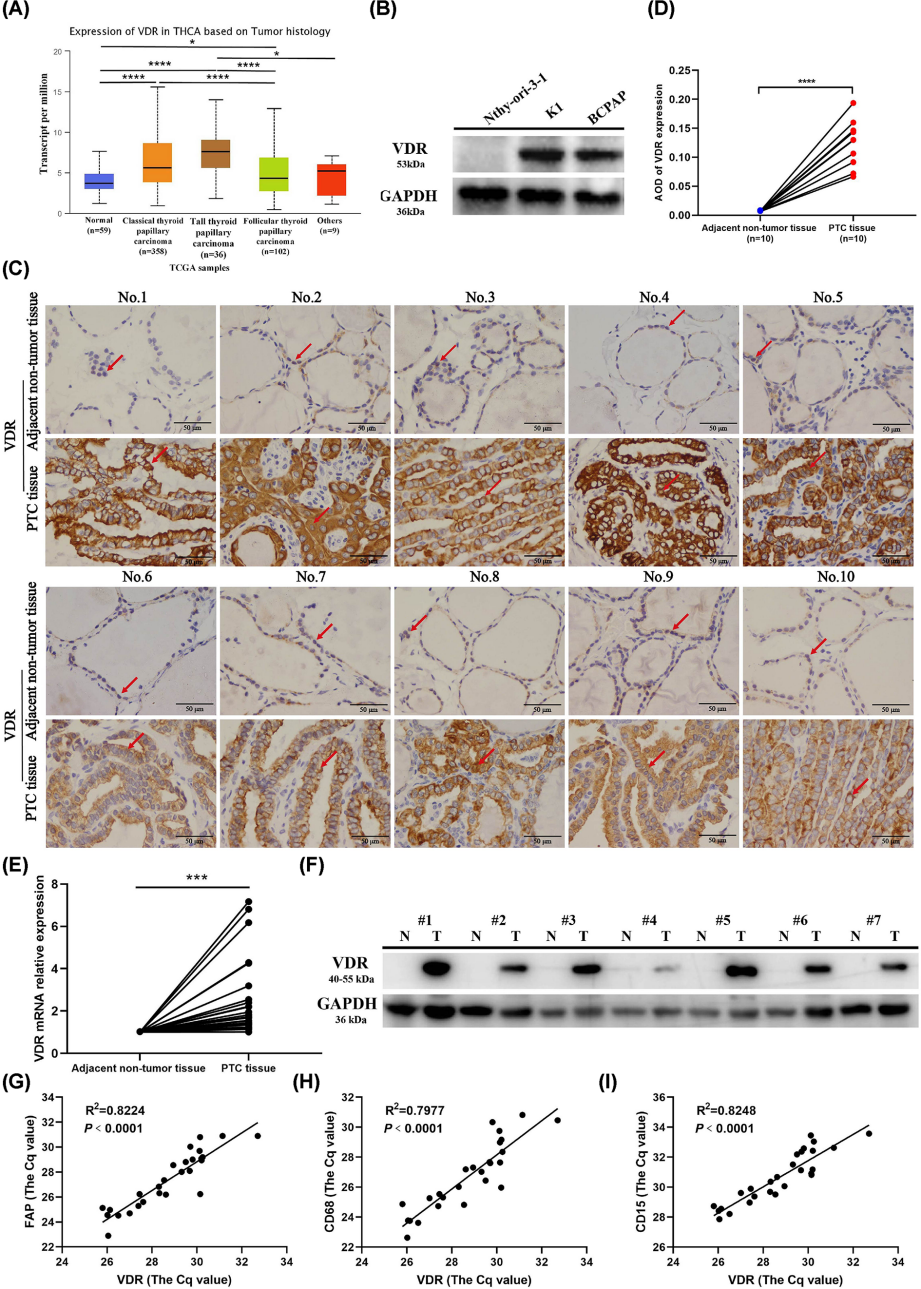
Experimental validation of VDR expression and the correlation with immune infiltration in PTC (**A**) VDR mRNA expression was higher in classical, tall and follicular thyroid papillary carcinoma than normal tissues classified by tumor histology based on the UALCAN database. (**B**) The higher expression of VDR in PTC cell lines (K1, BCPAP) when compared with normal thyroid cell line (Nthy-ori-3-1) was confirmed by Western blot analysis. (**C**) Immunohistochemical staining of VDR in the PTC tissues and adjacent non-tumor tissues (*n*=10), magnification ×400; Scale bar: 50 µm. The brown color indicated by red arrow represents positive staining. (**D**) Quantification of VDR expression by ImageJ software. (**E**) The average mRNA relative expression of VDR in PTC tissues and adjacent non-tumor tissues examined by qRT-PCR (*n*=27). (**F**) The representative protein expression of VDR in PTC tissues and adjacent non-tumor tissues examined by Western blot (*n*=27). The others were presented in Supplementary Figures S6 and S7; N: adjacent non-tumor tissue; T: PTC tissue. (**G–I**) Correlation analysis between the expression of VDR and FAP (cancer-associated fibroblast marker), CD68 (macrophage marker), and CD15 (neutrophil marker) in PTC tissues based on the *C*q values measured by qRT-PCR; *P<0.05, ***P<0.001, ****P<0.0001.

To determine the expression of VDR in thyroid carcinoma, firstly, we used the UALCAN platform to explore the mRNA expression of VDR in THCA based on tumor histology. The results showed that the expression of VDR was up-regulated in classical thyroid papillary carcinoma, tall thyroid papillary carcinoma and follicular thyroid papillary carcinoma (Figure 8A). The elevated expression of VDR was verified in PTC cell lines including K1 and BCPAP cells when compared with normal thyroid cell line Nthy-ori-3-1 (Figure 8B and Supplementary Figure S4 and S5). VDR was expressed in the nucleus in adjacent non-tumor tissues, but mainly located in the cytoplasm and cell membrane and showed stronger staining in PTC tissues (Figure 8C,D). In addition, VDR expression was increased in PTC tissues compared with matched adjacent non-tumor tissues at the mRNA and protein levels (Figure 8E,F and Supplementary Figures S6 and S7). In order to validate the correlation between VDR expression and immune infiltration, the expression of VDR and cell markers including FAP (cancer-associated fibroblast marker), CD68 (macrophage marker) and CD15 (neutrophil marker) in PTC tissues was examined by qRT-PCR [44–47]. The results showed that VDR expression was positively correlated with the expression of FAP, CD68 and CD15 in PTC tissues (Figure 8G–I).

### Knockdown of VDR promoted proliferation and migration of PTC cells

**Figure 9:**
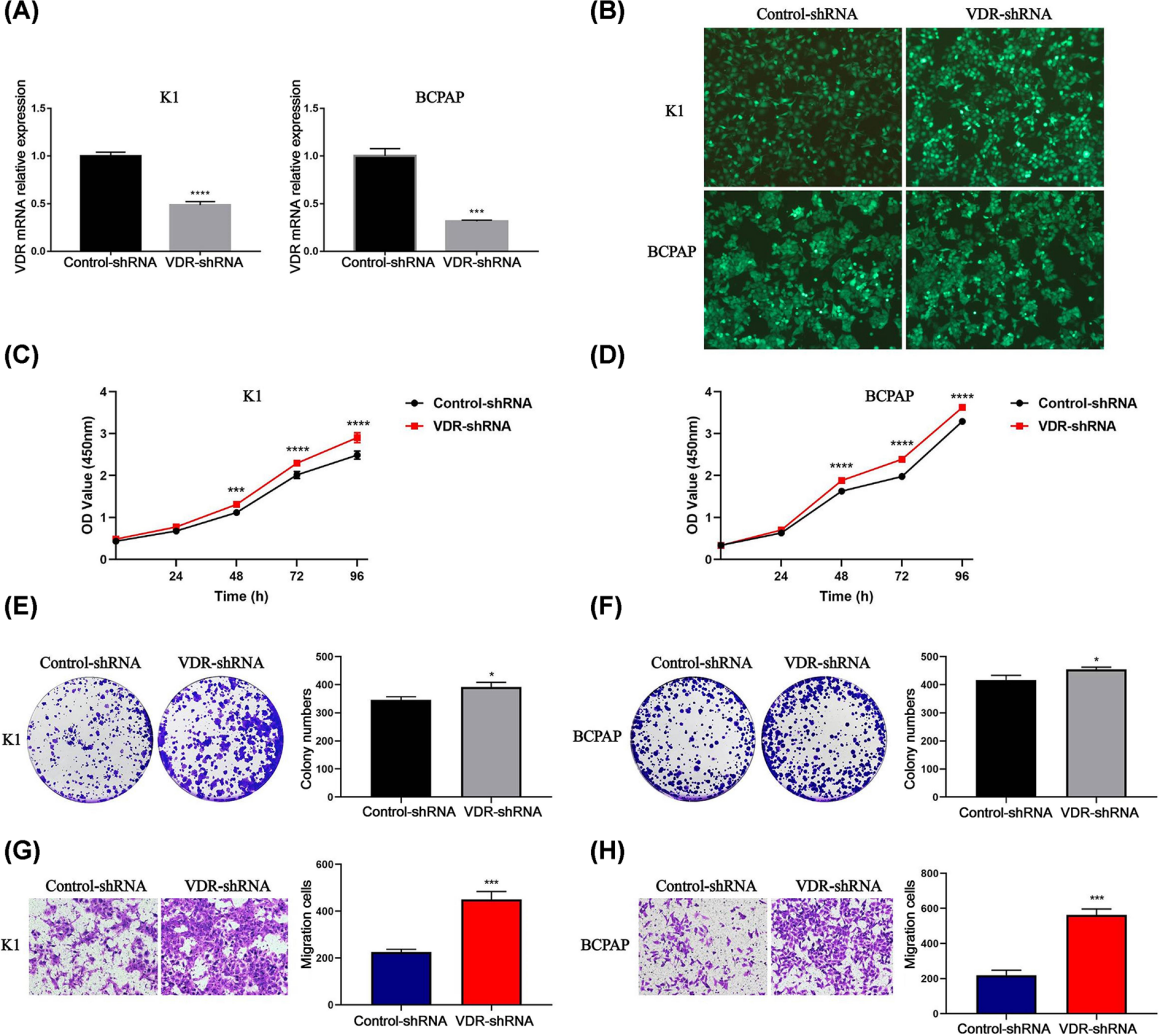
Knockdown of VDR promoted proliferation and migration of PTC cells (**A**) The mRNA knockdown efficiency of VDR by lentivirus in K1 and BCPAP cells was examined through qRT-PCR. (**B**) Green fluorescence indicated virus-infected K1 and BCPAP cells (>95%). (**C,D**) CCK-8 assay and (**E,F**) colony formation assay showed that cell proliferation was increased in the VDR-shRNA group than Control-shRNA group. (**G,H**) Transwell assay showed that the number of migration cells were higher in the VDR-shRNA group compared with Control-shRNA group. Each experiment was repeated three times; *P<0.05, **P<0.01, ***P<0.001, ****P<0.0001.

To determine the role of VDR in thyroid carcinoma cells, the expression levels of VDR in K1 and BCPAP cells were down-regulated by using lentivirus and cell proliferation and migration was examined. As shown in Figure 9A,B, the efficiency of VDR knockdown was approximately 50–70%. CCK-8 assay showed that VDR knockdown promoted K1 and BCPAP cell proliferation (Figure 9C,D). These results were confirmed by colony formation assay (Figure 9E,F). Transwell assay showed that the knockdown of VDR accelerated K1 and BCPAP cell migration (Figure 9G,H). These results indicate that VDR suppresses proliferation and migration of PTC cells.

### Overexpression of VDR inhibited proliferation and migration of PTC cell lines

**Figure 10:**
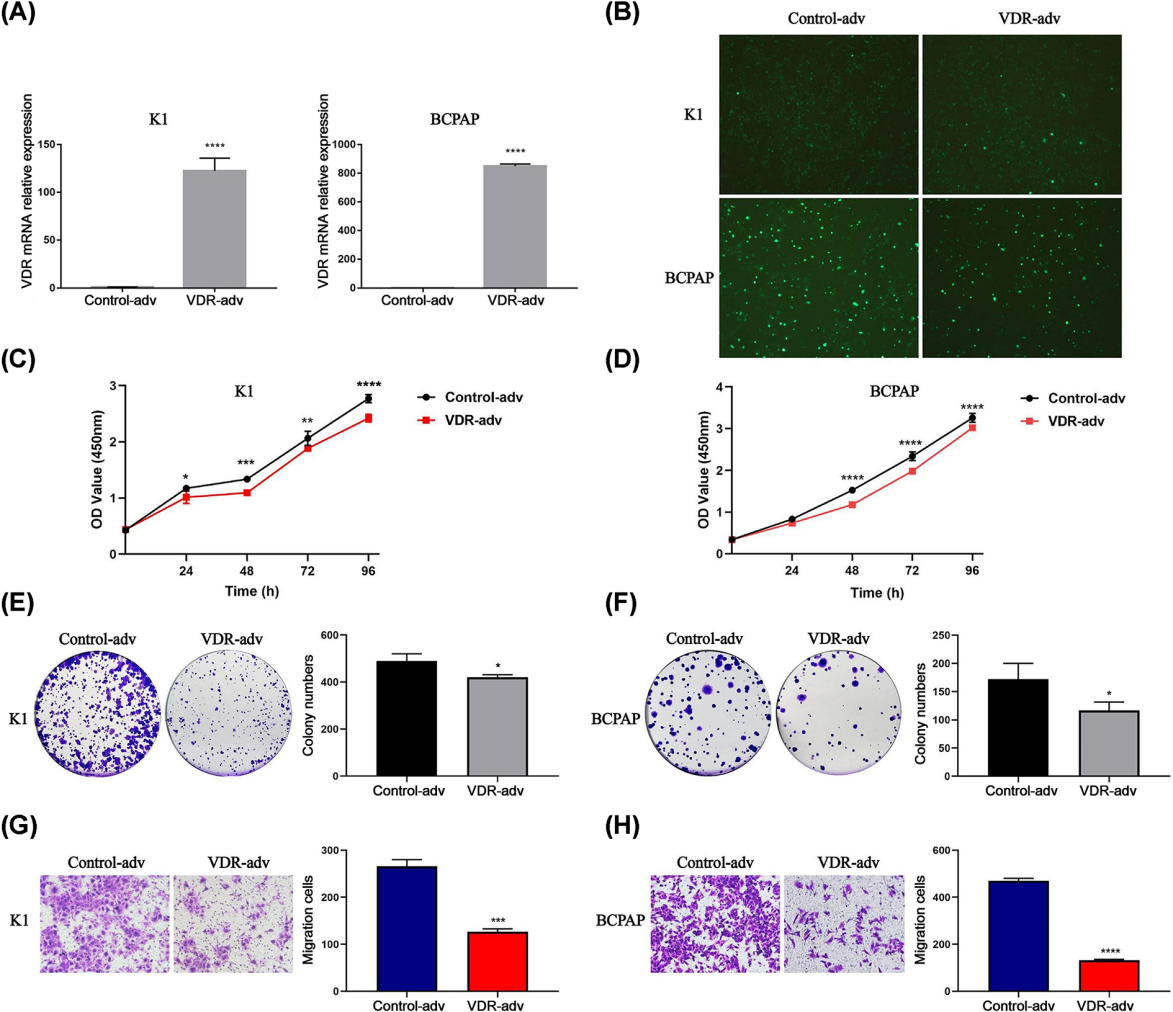
Overexpression of VDR inhibited proliferation and migration of PTC cell lines (**A**) The overexpression efficiency of VDR by adenovirus in K1 and BCPAP cells were determined by qRT-PCR at the transcriptional level. (**B**) The transfection efficiency of VDR was >90% (green fluorescence). (**C,D**) CCK-8 assay and (**E,F**) colony formation assay demonstrated that cell proliferation was suppressed in the VDR-adv group of K1 and BCPAP cells. (**G,H**) Transwell assay exhibited fewer migration cells in the VDR-adv group. Each experiment was repeated three times; *P<0.05, **P<0.01, ***P<0.001, ****P<0.0001.

In order to further confirm the suppressive role of VDR in tumor, VDR was overexpressed by adenovirus in K1 and BCPAP cells. As shown in Figure 10A, the mRNA expression levels of VDR were both increased by more than 100 folds in K1 and BCPAP cells. The transfection efficiency of VDR was over 90% (green fluorescence) and the virus-infected cells grew well (Figure 10B). The results indicated that VDR overexpression reduced the proliferation in K1 and BCPAP cells through CCK-8 assay (Figure 10C,D) and colony formation assay (Figure 10E,F). Transwell assay also showed that up-regulated expression of VDR suppressed the migration of K1 and BCPAP cells (Figure 10G,H).

## Discussion

As far as we know, this is the first pan-cancer study to analyze VDR expression and the association with clinical outcomes, immune infiltration, tumor microenvironment and gene set enrichment based on online databases and R software across 33 types of human cancers. This comprehensive pan-cancer analysis revealed the potential role of VDR in tumor progression and tumor immunity.

The present study showed that the expression of VDR was down-regulated in 8 types of human cancers including COAD, KIRC, KIRP, PCPG, PRAD, READ, SKCM, and THYM when compared with adjacent normal tissues. In addition, VDR expression was up-regulated in 12 types of human cancers including BRCA, CESC, CHOL, ESCA, HNSC, KICH, LIHC, LUAD, OV, STAD, THCA, and UCEC The heterogeneity may be due to a relatively small number of normal tissues or different mechanisms of VDR in various tumors. There have been reported that the expression of VDR is decreased in colorectal adenocarcinoma and renal clear cell cancer and increased in breast invasive carcinoma, ovarian tumor, and thyroid papillary carcinoma [13,48–51]. Furthermore, the expression of VDR was affected by the degree of tumor differentiation. Previous studies have shown that VDR expression decreases with tumor dedifferentiation in esophageal adenocarcinoma and increased malignant degree in thyroid carcinoma [52,53]. Besides, VDR is abundantly expressed in cancer tissues with high levels of differentiation but has low or undetectable levels of expression in cancer tissues with moderate or low levels of differentiation in pancreatic carcinoma [28]. The present study also demonstrated that the expression of VDR varied between different pathological stages in HNSC, KICH, TGCT, and THCA. Especially in KICH and TGCT, VDR expression increased in the early stage but decreased in the late stage, which may be attributed to the compensatory role of VDR in early tumor progression and the lost of compensatory effect in the advanced stage.

Survival analysis showed that higher expression of VDR was associated with more favorable prognosis in CHOL, SKCM, KIRC, ESCA and OV. Similar results for CHOL have been reported in previous studies [54]. In contrast, higher expression of VDR was associated with poorer prognosis in LGG, GBM, LAML, KIRP, ACC, PAAD, UVM and THCA. Wang et al. have found that a lower expression of VDR is associated with worse outcomes in PAAD, and Salomón et al. have demonstrated that higher VDR expression is correlated with better survival in GBM [28,55]. Moreover, Huss et al. have reported high expression of VDR is a positive prognostic factor in BRCA [13]. The clinical outcomes of cancer patients are influenced by many confounding factors such as age, comorbidity, basic health status and degree of tumor malignancy, different studies may get inconsistent results due to different populations and baselines of patients. Although there have been inconsistent conclusions about the relationship between VDR expression levels and survival in some types of tumors, the present study revealed that genetic alterations of VDR were associated with significantly reduced survival in ACC, LUAD, and OV.

It is known that the immune system plays an essential role in cancer initiation and progression [56,57]. Integrated analyses of immune infiltration and immunomodulators in tumors have provided novel insights into understanding the effect of immune system on tumor growth [58–60]. Our results showed that VDR expression was positively correlated with infiltration of cancer associated fibroblasts, macrophages or neutrophils in 20, 12, and 10 types of human tumors respectively. The positive correlations of VDR expression with the expression of FAP (cancer-associated fibroblast marker), CD68 (macrophage marker), or CD15 (neutrophil marker) were experimentally verified in PTC tissues. Moreover, VDR expression was positively correlated with multifarious immunomodulators especially in GBM and TGCT in the present study. These results suggested that VDR may regulate immune response during tumor progression. Previous studies have focused on the cancer-promoting role of cancer associated fibroblasts; however, latest studies have suggested tumor-inhibitory capabilities for cancer associated fibroblast subpopulations based on the cellular heterogeneity analysis [61,62]. It has been reported that high expression of VDR in tumor stromal fibroblasts was correlated with favorable prognosis in colorectal cancer [14]. In addition, macrophages and neutrophils are mostly from VDR positive hematopoietic precursor [63]. According to the degree of activation, macrophages are divided into M1 macrophages and M2 macrophages. M1 macrophages promote tumor eradication while M2 macrophages contribute to tumor progression. Similarly, neutrophils manifested anti-tumor and pro-tumor properties. Szczerba et al. have shown that neutrophils accelerate cell cycle progression and extend the metastatic potential in circulating tumor cells while Governa et al. have demonstrated that neutrophils consolidate the responsiveness of CD8+ T cells and facilitate antineoplastic immunity in colorectal cancer [64,65]. Due to immune cells such as macrophages have been shown to have opposing functions in regulating cancer progression, the definitive roles of VDR in tumor immunity remains to be further elucidated.

Tumor microenvironment, which is mainly composed of immune and stromal components, is associated with progression and prognosis of tumors [66,67]. A variety of algorithms have been exploited to estimate the components of immune and stromal cell such as ESTIMATE, TIMER, and CIBERSORT [68–70], among which ESTIMATE algorithm is a reliable and an effective method that has been widely implemented [71,72]. The higher ImmuneScore, StromalScore and ESTIMATEScore are, the higher the stromal and immune cell proportion and the less tumor purity in the tumor microenvironment are [73]. High tumor purity is considered to be associated with high risk or poor clinical outcomes in some cancers such as skin cutaneous melanoma and colorectal cancer [74,75]. The present study showed that VDR expression had a positive relationship with StromalScore, ESTIMATEScore or ImmuneScore while it was negatively correlated with TumorPurity in 24 types of human cancers including TGCT, GBM, LAML, DLBC, LUSC, LGG, SARC, PAAD, CHOL, UCS, PRAD, OV, LUAD, SKCM, UVM, THCA, BLCA, MESO, HNSC, PCPG, LIHC, BRCA, CESC, and UCEC. The results from the present studies indicate that VDR may participate in regulating the proportion of immune and stromal cells in tumor microenvironment. Therefore, it may be possible to use VDR agonists to reshape the tumor microenvironment for anti-tumor purposes.

VDR, as a transcription factor, is widely expressed in multiple tumor tissues and regulates the expression of a variety of downstream signaling molecules. Gene enrichment analysis data in the present study revealed that VDR may potentially influence immune regulation including Th1/Th2 cell differentiation, T/B receptor/chemokine signaling pathway, cytokine–cytokine receptor interaction, complement and coagulation cascades, or NK cell-mediated cytotoxicity in tumors. By analyzing the known VDR transcription factor binding sites, Singh et al. have found that VDR peaks are enriched for SNPs linked to immune phenotypes, indicating that the immuno-modulatory functions are the main effect of VDR [76]. However, how VDR affects tumor progression through immunity remains to be determined. Our studies also showed that VDR negatively correlated genes were involved in metabolism-related pathways encompassing protein export, RNA transport, carbon metabolism, glyoxylate and dicarboxylate metabolism, tyrosine metabolism, arginine and proline metabolism, or fatty acid metabolism in cancer tissues, suggesting that VDR inhibits tumor growth possibly via suppressing energy metabolism.

Thyroid carcinoma is the most common malignant neoplasm in the endocrine system. The higher expression of VDR in PTC was confirmed in the present study when compared with normal thyroid and the result was consistent with the UALCAN database. The immunohistochemical results showed that VDR was mainly expressed in the cytoplasm and cell membrane in PTC tissues. The cytoplasmatic or membranous expression of VDR has also been found in other tumors such as breast cancer and ovarian cancer [13,77]. It is now believed that VDR localized in the nucleus regulates gene transcription (genomic response), while VDR localized in the cytoplasm and cell membrane plays a role in rapid signal transduction (non-genomic response) [78,79]. The proliferation and migration of K1 and BCPAP cells were promoted after VDR expression was down-regulated by lentivirus. Moreover, the proliferation and migration of K1 and BCPAP cells were significantly inhibited after VDR expression was overexpressed by adenovirus. Other studies *in vitro* have also indicated that VDR suppresses cell proliferation in colon cancer and VDR participates the inhibition of epithelial-mesenchymal transition in human laryngeal squamous cell carcinoma [80,81]. However, our survival analysis manifested that up-regulation of VDR was associated with poor survival in thyroid carcinoma which was sync with the research performed by Choi et al. based on public multigenomics data [21]. The discrepancy between *in vitro* experiments and clinical data is affected by multiple factors. Tumors are in a more complex environment in the human body and the results of clinical studies are affected by individual differences.

Nevertheless, there are still some limitations. The present study was mainly based on public databases and preliminary cell experiments, further *in vivo* experiments and prospective cohorts are required to gain a deeper understanding of the role and molecular mechanisms of VDR in tumor progression especially in the regulation of immune response and tumor microenvironment.

## Conclusion

This first pan-cancer analysis of VDR indicates that VDR may serve as a prognostic biomarker and VDR is positively associated with immune infiltration as well as stromal or immune components in the tumor microenvironment in multiple human cancers. VDR positively correlated genes are enriched in immune cell function pathways while negatively correlated genes are involved in energy metabolism pathways in the top 9 highly lethal tumors. The expression of VDR is increased in PTC cells and tissues and VDR inhibits the proliferation and migration of PTC cells. In addition, the positive correlations between VDR expression and the expression of cancer-associated fibroblast, macrophage, and neutrophil markers are experimentally verified in PTC. The present study provides new insights into the potential roles of VDR in tumor progression and tumor immunity in human cancers, thus laying the foundation for further studies.

## Supplementary Material

Supplementary Figures S1-S7 and Table S1

## Data Availability

The data generated during and/or analyzed during the current study are available from the corresponding author on reasonable request.

## Abbreviations

ACC: adrenocortical carcinoma
ATCC: American Type Culture Collection
B2M: β2-microglobulin
BLCA: bladder urothelial carcinoma
BRCA: breast invasive carcinoma
CCK-8: cell counting kit-8
CCL2: C-C motif chemokine ligand 2
CCL22: C-C chemokine ligand 22
CCR1: C-C motif chemokine receptor 1
CCR2: C-C motif chemokine receptor 2
CCR5: C-C motif chemokine receptor 5
CCTCC: China Center for Type Culture Collection
CESC: cervical squamous cell carcinoma and endocervical adenocarcinoma
CHOL: cholangiocarcinoma
COAD: colon adenocarcinoma
CPTAC: Clinical Proteomics Tumor Analysis Consortium
CSF1R: colony stimulating factor 1 receptor
CXCL16: C-X-C motif chemokine ligand 16
DAB: diaminobenzidine
DFI: disease free interval
DLBC: lymphoid neoplasm diffuse large B-cell lymphoma
DMEM: Dulbecco’s Modified Eagle Medium
DSS: disease specific survival
ESCA: esophageal carcinoma
FAP: fibroblast activation protein
FBS: fetal bovine serum
GBM: glioblastoma multiforme
GSEA: gene set enrichment analysis
GTEx: Genotype-tissue expression
HLA-A: human leukocyte antigen-A
HNSC: head and neck squamous cell carcinoma
IHC: immunohistochemistry
IL2RA: interleukin-2 α-chain receptor
KEGG: Kyoto Encyclopedia of Genes and Gnomes
KICH: kidney chromophobe
KIRC: kidney renal clear cell carcinoma
KIRP: kidney renal papillary cell carcinoma
LAML: acute myeloid leukemia
LGALS9: galectin-9
LGG: brain lower grade glioma
LIHC: liver hepatocellular carcinoma
LUAD: lung adenocarcinoma
LUSC: lung squamous cell carcinoma
MESO: mesothelioma
MHCs: major histocompatibility complexes
MOI: multiplicity of infection
NK cell: natural killer cell
OD: optical density
OS: overall survival
OV: ovarian serous cystadenocarcinoma
PAAD: pancreatic adenocarcinoma
PCPG: pheochromocytoma and paraganglioma
PFS: progression free survival
PRAD: prostate adenocarcinoma
PTC: papillary thyroid cancer
qRT-PCR: quantitative real-time polymerase chain reaction
READ: rectum adenocarcinoma
RESM: RNA-Seq by Expectation-Maximization
SARC: sarcoma
SKCM: skin cutaneous melanoma
STAD: stomach adenocarcinoma
TAPBP: tapasin-binding protein
TCGA: The Cancer Genome Atlas
TGCT: testicular germ cell tumors
THCA: thyroid carcinoma
THYM: thymoma
TME: tumor microenvironment
TNFRSF13: tumor necrosis factor TNF receptor superfamily member 13
Treg: T regulatory cell
UCEC: uterine corpus endometria carcinoma
UCS: uterine carcinosarcoma
UVM: uveal melanoma
VDR: vitamin D receptor
