# Supplementary figures and images for: VDR is a potential prognostic biomarker and positively correlated with immune infiltration: a comprehensive pan-cancer analysis with experimental verification
Source: Biosci Rep. 2024 Apr 30;44(5):BSR20231845. doi: 10.1042/BSR20231845 (PMC11065647; doi:10.1042/BSR20231845)

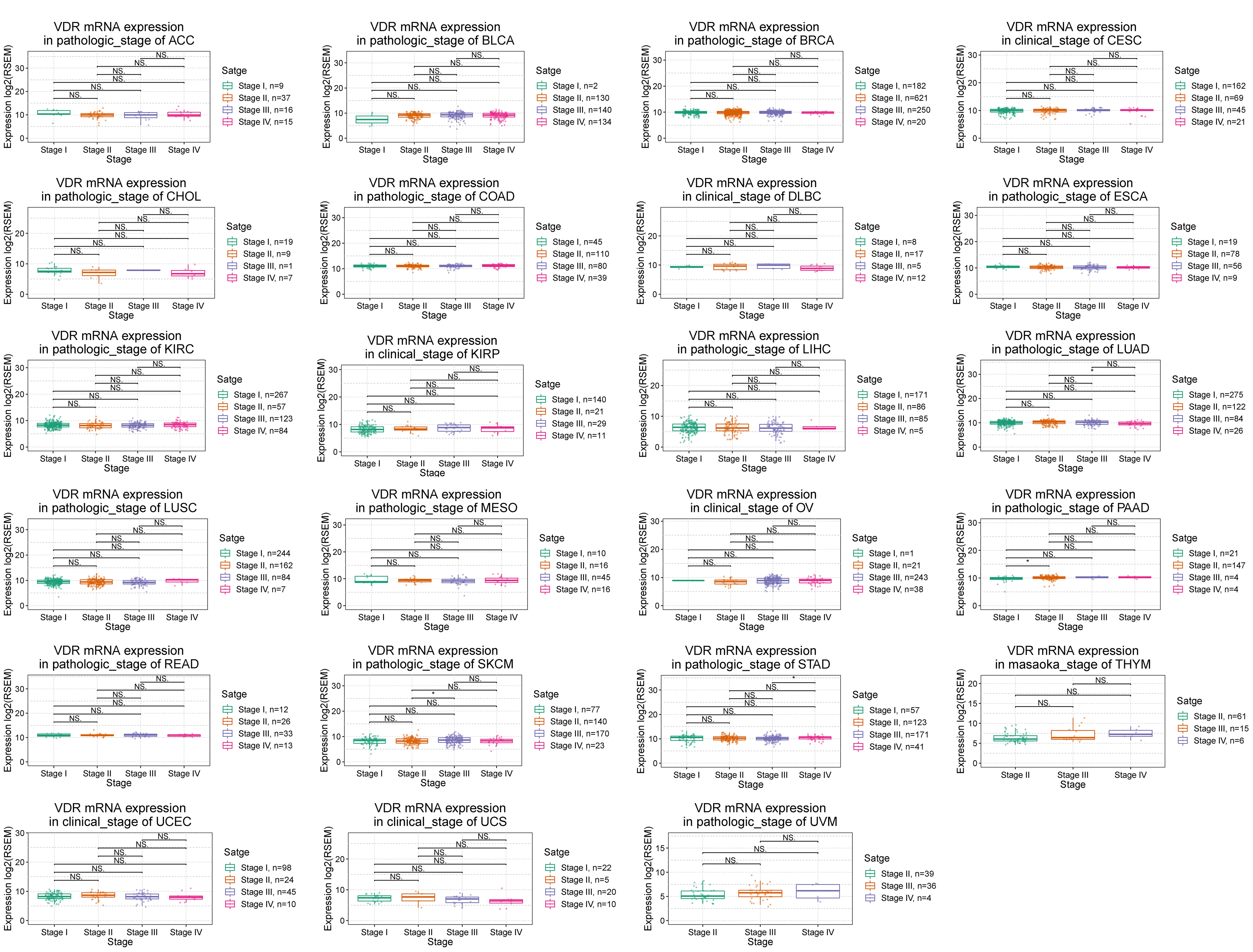

Supplement: Supplementary Figures S1-S7 and Table S1 [file BSR-2023-1845_supp.zip › BSR-2023-1845_suppS1.jpg]

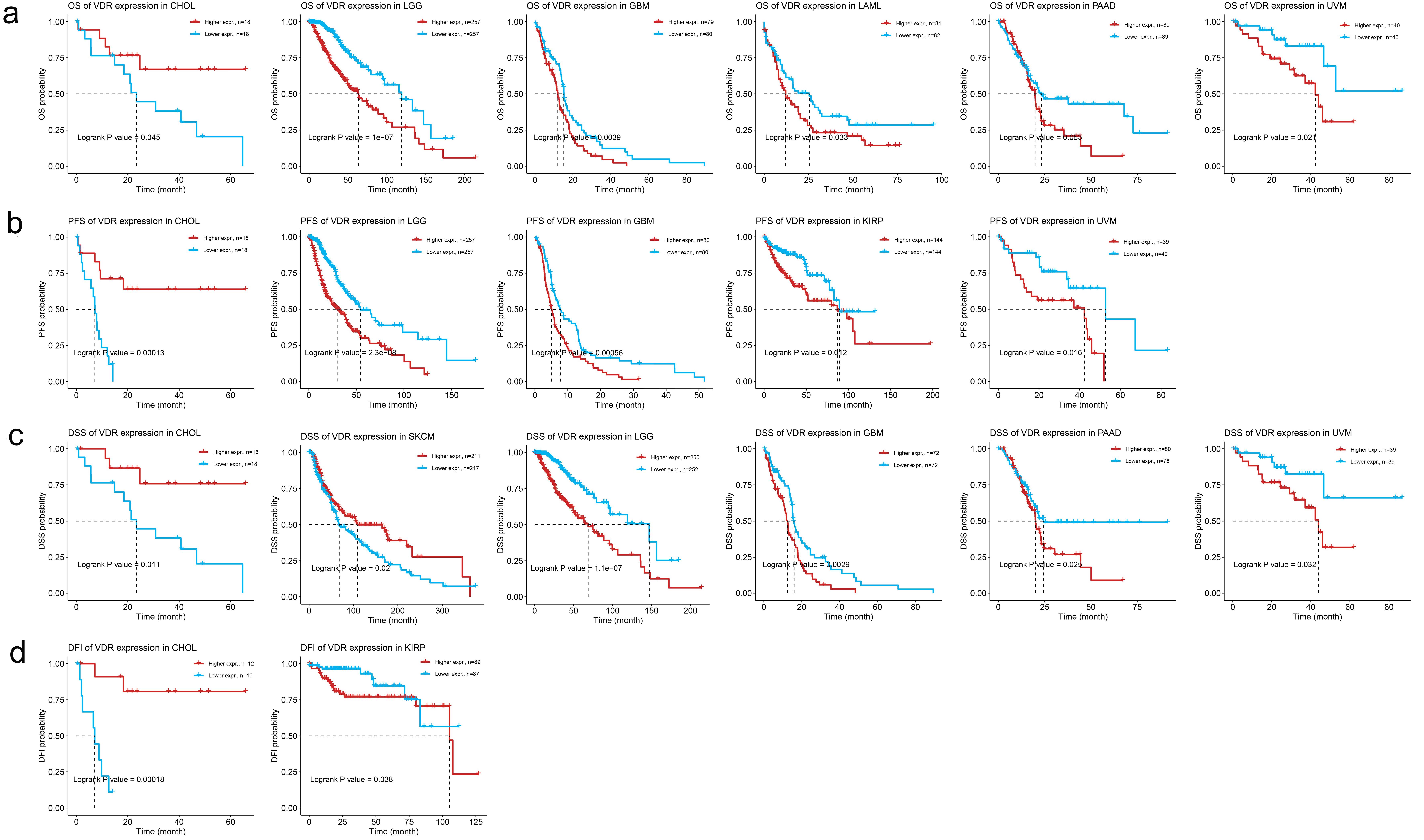

Supplement: Supplementary Figures S1-S7 and Table S1 [file BSR-2023-1845_supp.zip › BSR-2023-1845_suppS2.jpg]

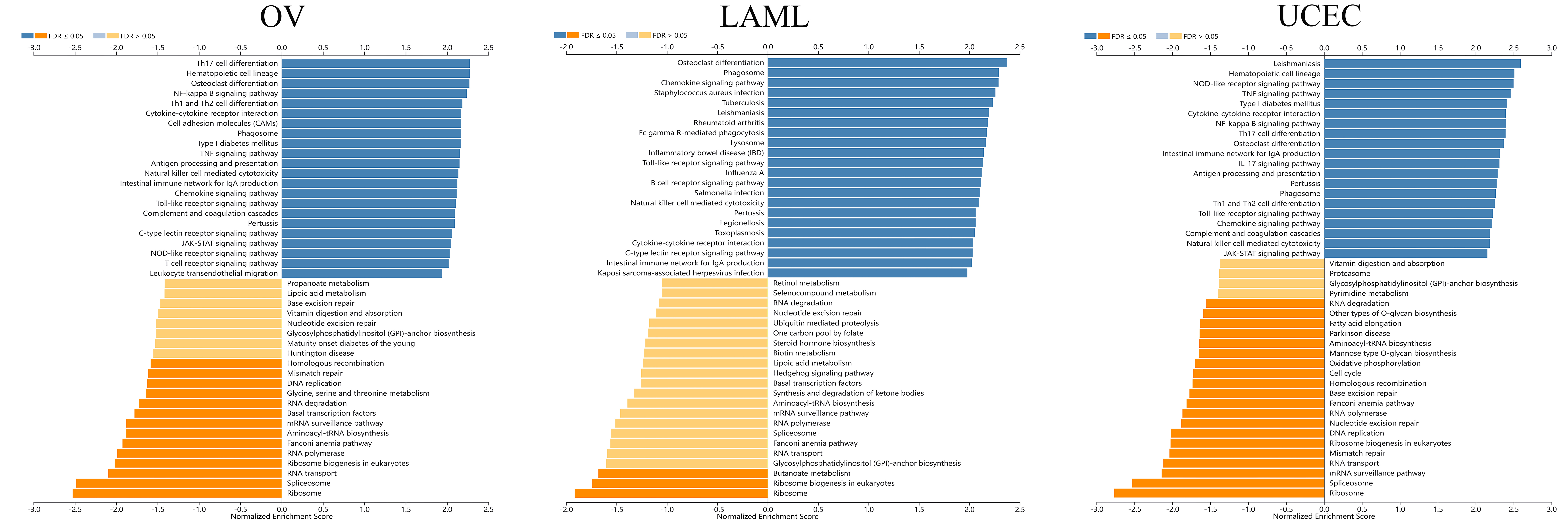

Supplement: Supplementary Figures S1-S7 and Table S1 [file BSR-2023-1845_supp.zip › BSR-2023-1845_suppS3.jpg]

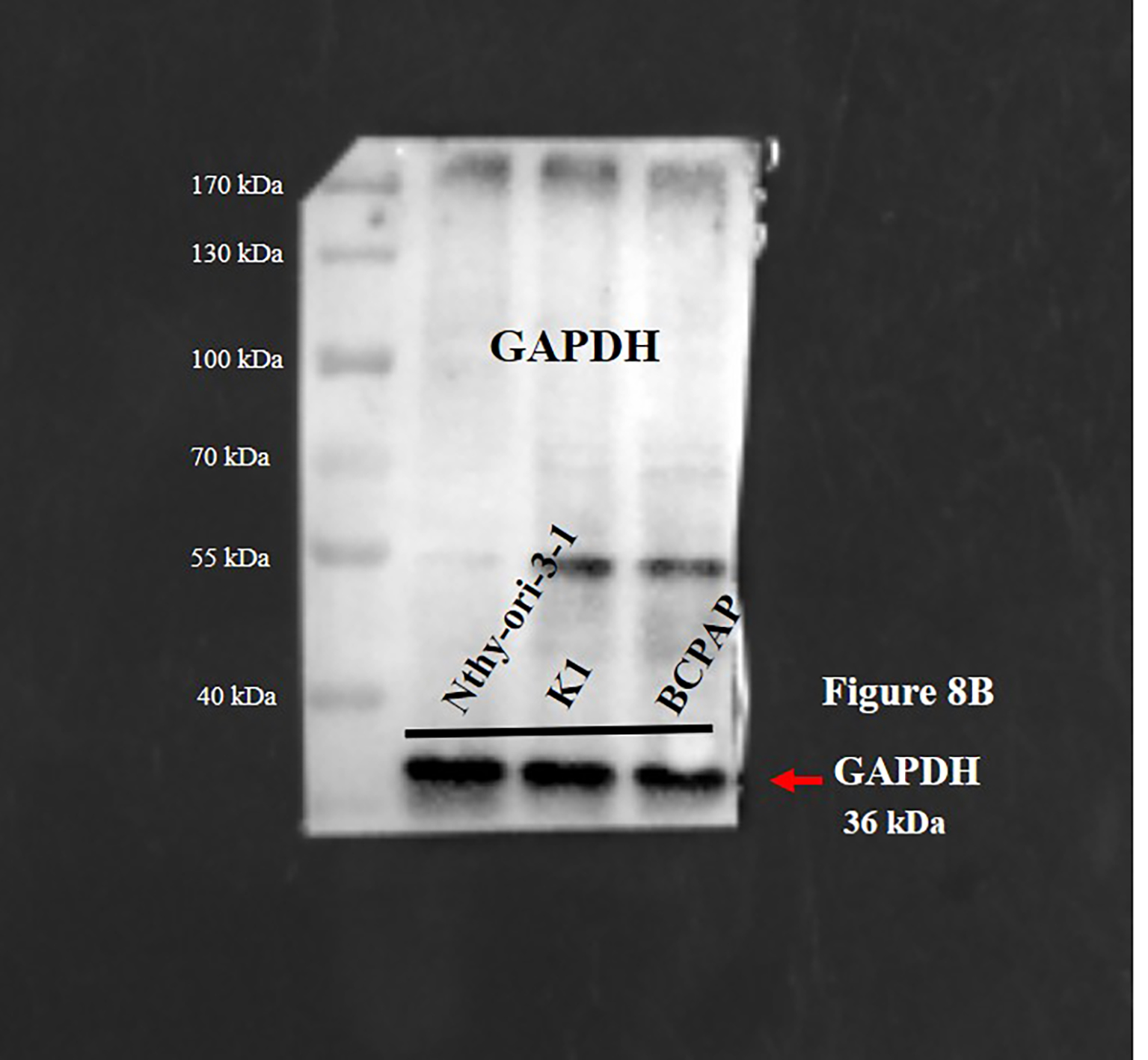

Supplement: Supplementary Figures S1-S7 and Table S1 [file BSR-2023-1845_supp.zip › BSR-2023-1845_suppS4.jpg]

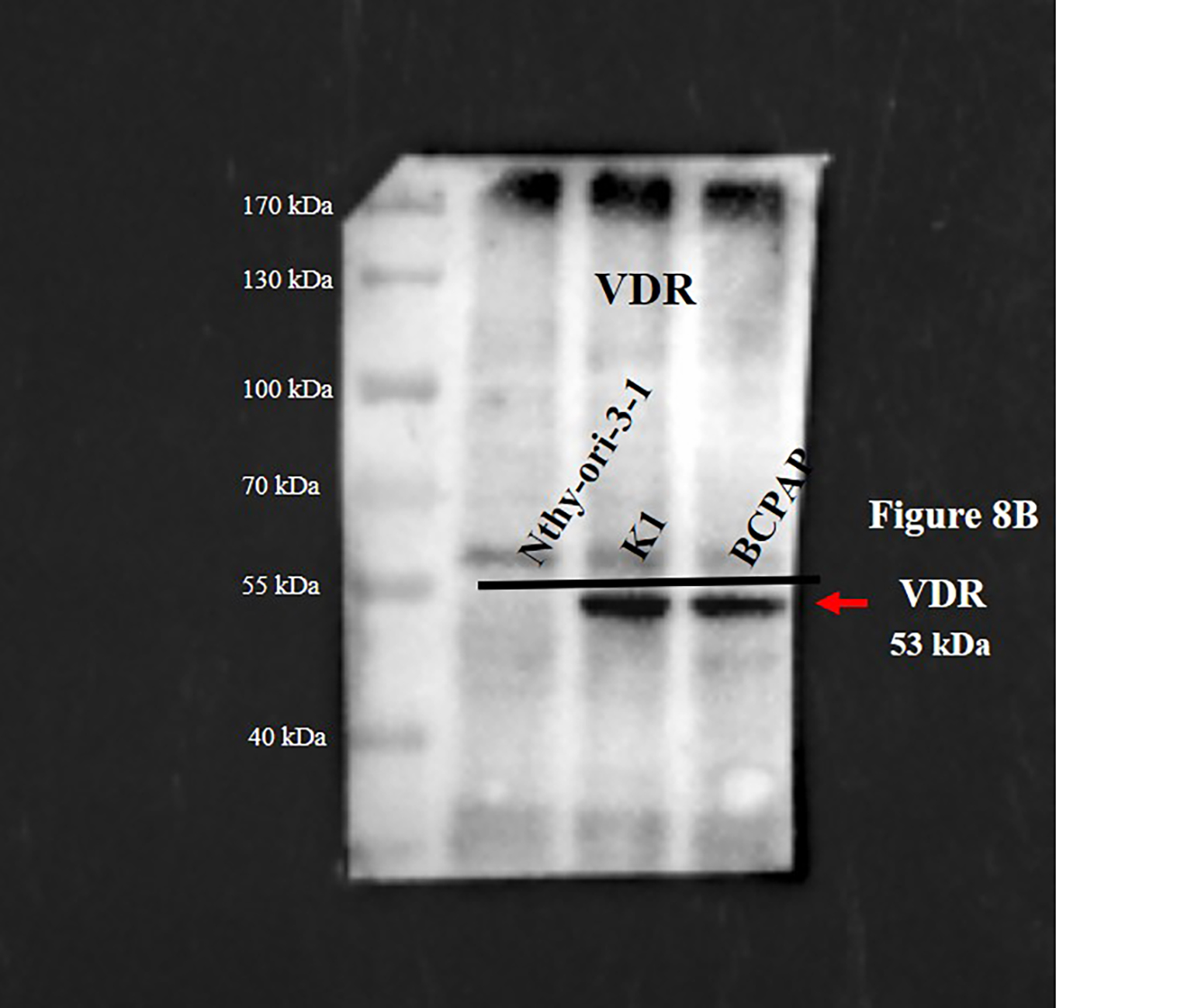

Supplement: Supplementary Figures S1-S7 and Table S1 [file BSR-2023-1845_supp.zip › BSR-2023-1845_suppS5.jpg]

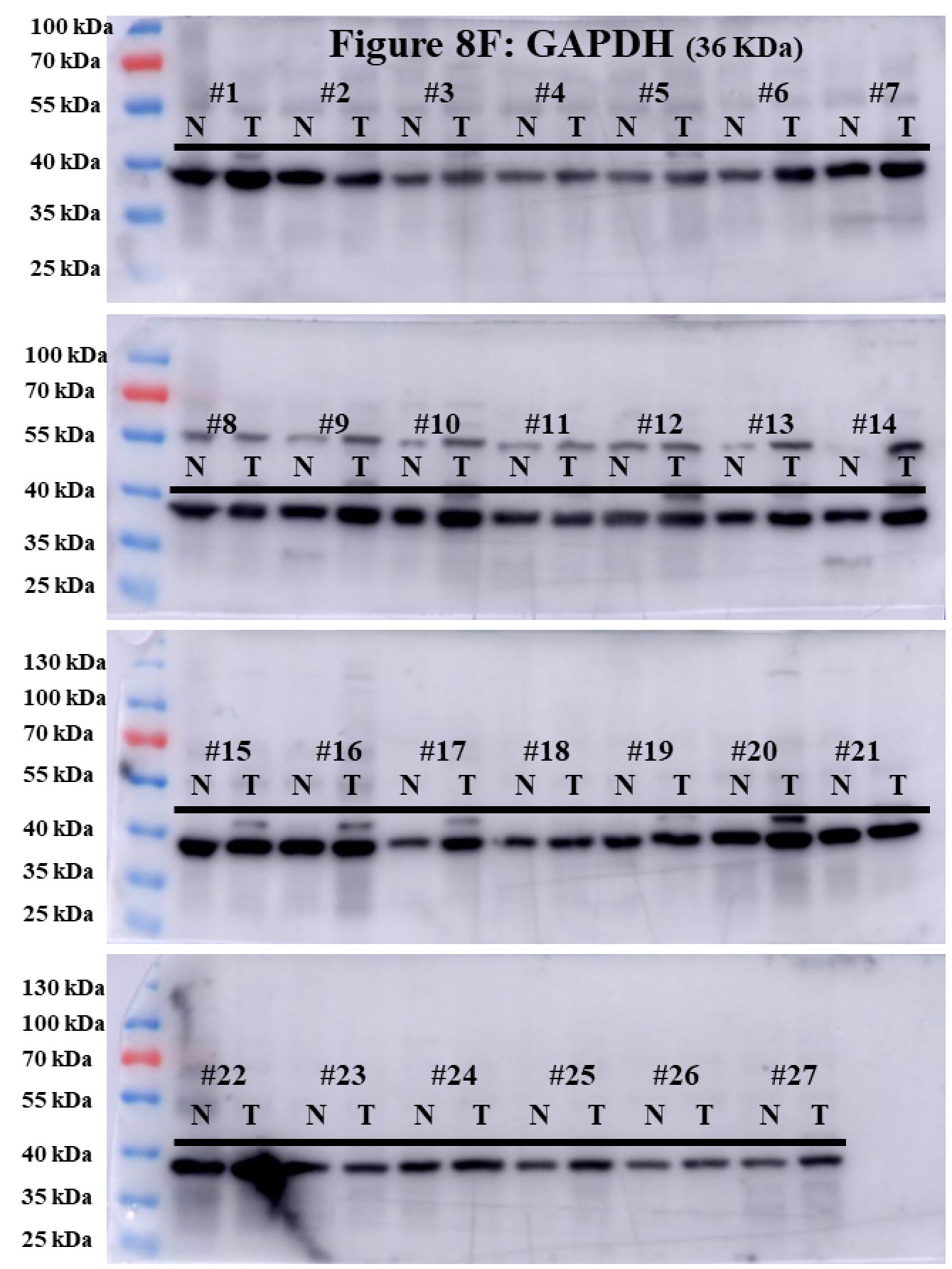

Supplement: Supplementary Figures S1-S7 and Table S1 [file BSR-2023-1845_supp.zip › BSR-2023-1845_suppS6.jpg]

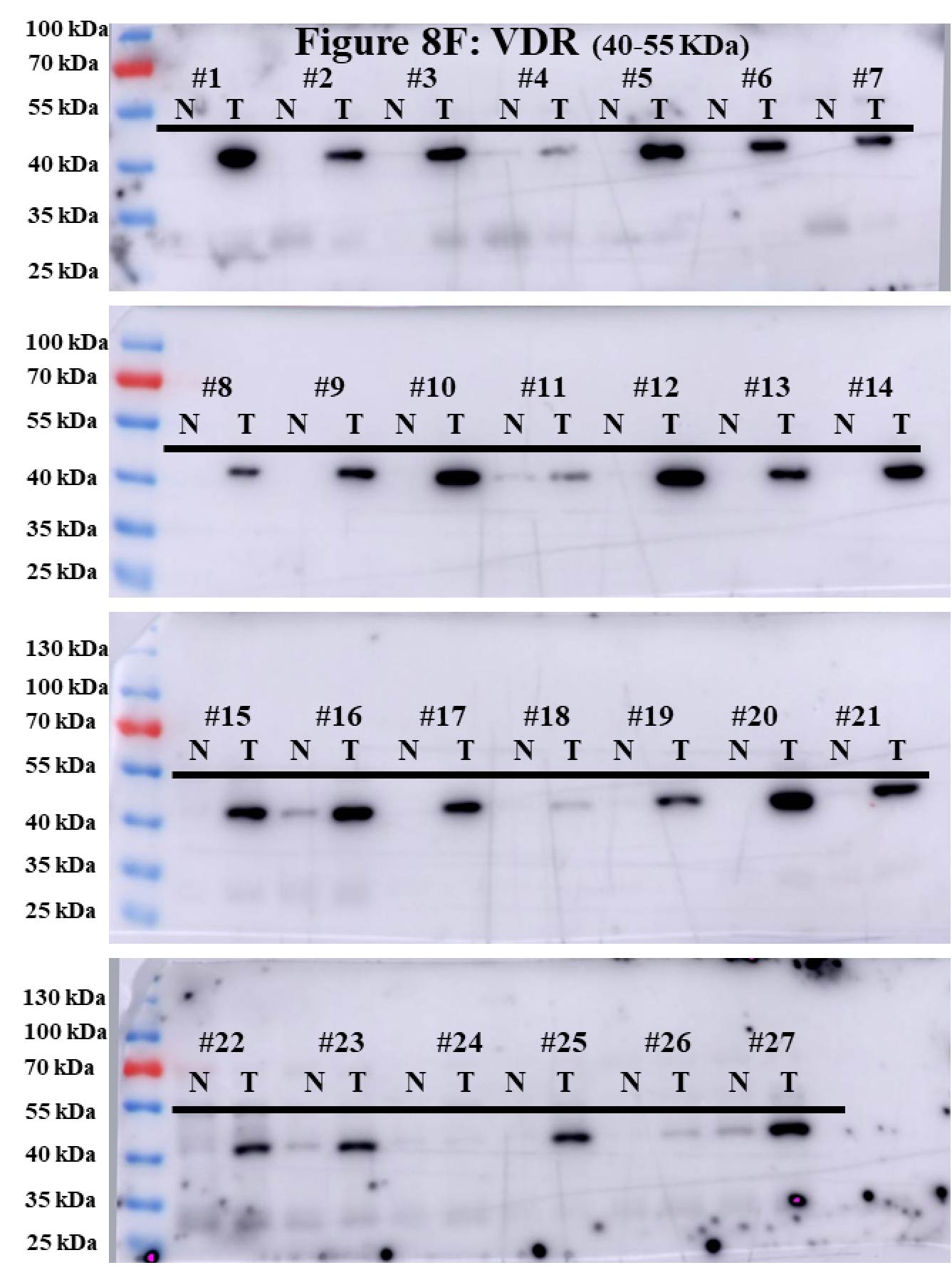

Supplement: Supplementary Figures S1-S7 and Table S1 [file BSR-2023-1845_supp.zip › BSR-2023-1845_suppS7.jpg]

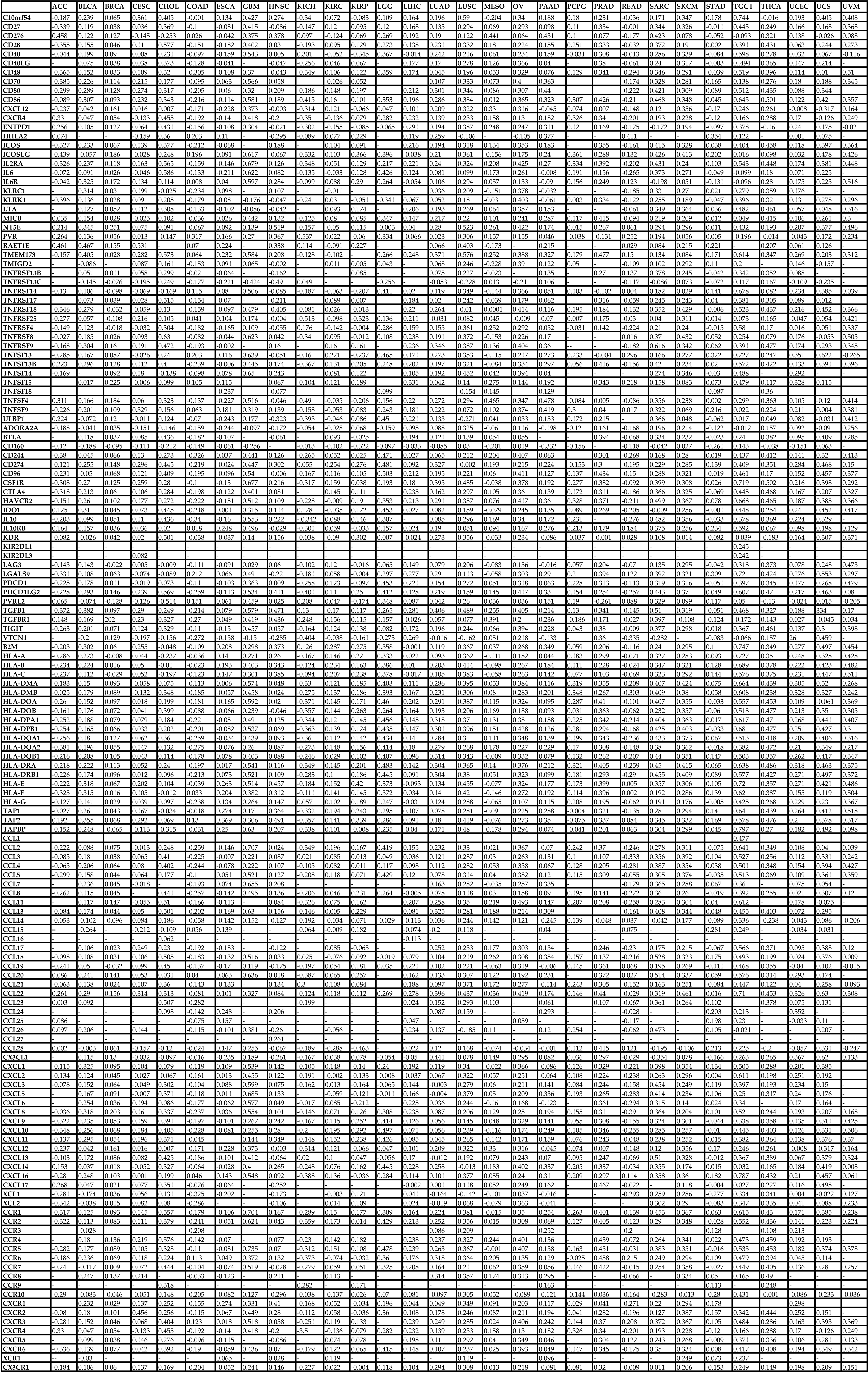

Supplement: Supplementary Figures S1-S7 and Table S1 [file BSR-2023-1845_supp.zip › BSR-2023-1845_suppSt1.jpg]
